# Supplementary material for: Systematic discovery of protein interaction interfaces using AlphaFold and experimental validation
Source: Mol Syst Biol. 2024 Jan 15;20(2):75–97. doi: 10.1038/s44320-023-00005-6 (PMC10883280; doi:10.1038/s44320-023-00005-6)
Supplement: Supplementary file 1 — Appendix text and figures [file 44320_2023_5_MOESM1_ESM.pdf]

Appendix

Systematic discovery of protein interaction interfaces using AlphaFold and experimental validation

Chop Yan Lee[1,†], Dalmira Hubrich[1,†], Julia K. Varga[2,†], Christian Schäfer[1], Mareen Welzel[1], Eric Schumbera[3], Milena Đokić[1], Joelle M. Strom[1], Jonas Schönfeld[1], Johanna L. Geist[1], Feyza Polat[1], Toby J. Gibson[4], Claudia Isabelle Keller Valsecchi[1], Manjeet Kumar[4], Ora Schueler-Furman[2,*], Katja Luck[1,**]

Affiliations

1 Institute of Molecular Biology (IMB) gGmbH, 55128 Mainz, Germany.

2 Department of Microbiology and Molecular Genetics,Institute for Biomedical Research Israel-Canada, Faculty of Medicine, The Hebrew University of Jerusalem, Jerusalem 9112001, Israel.

3 Institute of Molecular Biology (IMB) gGmbH, 55128 Mainz, Germany. Current address: Computational Biology and Data Mining Group Biozentrum I 55128 Mainz, Germany.

4 Structural and Computational Biology Unit, European Molecular Biology Laboratory, Heidelberg, 69117, Germany.

*Corresponding author. Tel: +972-2-675-7094, E-mail: ora.furman-schueler@mail.huji.ac.il

**Corresponding author. Tel: +49-(0)6131-3921440, E-mail: k.luck@imb-mainz.de

†These authors contributed equally to this work.

Table of content

Appendix Text S1. Summary of observations from the manual inspection of AlphaFold models generated from fragmentation approach on PPIs connecting NDD proteins.

Appendix Figure S1. Benchmarking of AF on DMI interfaces using minimal interacting regions.

Appendix Figure S2. Benchmarking and application of AF for DMI interface prediction using minimal interacting fragments.

Appendix Figure S3. Effect of protein fragment extensions on the accuracy of AF predictions.

Appendix Figure S4. Effect of protein fragment extensions on the accuracy of AF predictions.

Appendix Figure S5. Comparison of AF v2.2 and v2.3 prediction performance.
Appendix Figure S6. Performance of different metrics derived from structural models when benchmarking AF v2.3 for DMI predictions.
Appendix Figure S7. Expression and BRET50 plots for TRIM37-PNKP and ESRRG-PSMC5.
Appendix Figure S8. Structural models, expression, and BRET50 plots for STX1B-FBXO28 and STX1B-VAMP2.
Appendix Figure S9. Structural models, expression, and BRET50 plots for PEX3-PEX19 and PEX3-PEX16.
Appendix Figure S10. Expression and BRET50 plots for SNRPB-GIGYF1.

Appendix Text S1. Summary of observations from the manual inspection of AlphaFold models generated from fragmentation approach on PPIs connecting NDD proteins.

run14: PLP1-MFF

Top prediction involves an ordered region from PLP1 and a disordered fragment from MFF, with a model confidence of 0.75. Looking at the predicted model, the peptide is tilted at an angle to the bundle of helices of PLP1, not like the usual coiled-coil interaction. No trend in increasing confidence with shorter fragments too. The interface does not look very convincing. While the disordered region in MFF is likely to be a functional motif, the 4-helix bundle domain in PLP1 that AF models it to bind to is known to be a transmembrane domain, so the binding site is actually buried inside the membrane. AF is also not very confident about the domain structure, especially for the parts that are at the membrane surface or outside of it. The prediction is likely wrong.

run17: PAX6-CSNK2A1

CSNK2A is a widely active kinase, involved in many processes. Overlapping fragments from PAX6 show trend of increasing confidence the shorter the fragment. CSNK2A1 is predicted to bind with its kinase domain (it doesn't really has anything else than the kinase domain) to a peptide in PAX6 which seems to be a good looking linear motif, i.e. conserved, not part of a folded domain as predicted by AF and predicted by AF to form an alpha helix. The motif though overlaps with a putative NLS. The PAX6 motif is predicted to bind clearly to a pocket that exists in N-lobe of the kinase domain at the bottom of it, away from the catalytic side. Digging deeper, I found a structure, 1JWH, that shows that this is the pocket that is bound by CSNK2B, the regulatory subunit, that interacts with the catalytic subunit to form an active holoenzyme. This, however, does not eliminate the possibility that the AF prediction is right since the peptide looks like a functional motif.

run18: PAX6-SET

Top prediction is ordered-ordered, PAX6 Homeodomain and SET NAP domain. The structure 6PAX shows the PAX domain consisting of two similar folds like the homeodomain bound to DNA but the three-helix bundles are not oriented in exactly the same way like in the homeodomain so I am having a hard time to see where the homeodomain would bind DNA; AF models the homeodomain interface with the NAP domain of SAP via a charged interface with a lot of positively charged residues on the homeodomain contacting a patch of negatively charged residues on the SAP domain. It could be that this patch of positively charged residues on the homeodomain would usually interact with the negatively charged backbone of DNA, but the predicted structure from AF looks interesting since the interface likely does not interfere with SET homodimerization (2E50).

run19: PAX6-TLK2

All predictions with >0.7 model confidence are paired with the Pkinase domain of TLK2 and they are all predicted to bind at the bottom of the beta barrel fold (N-lobe) of the kinase domain. However, almost all peptides come from very different regions in PAX6, no recurrent predictions here.

When looking at the motif pLDDT metric then top predictions also involve two distinct motifs predicted to bind to the long helices in TLK2. However, AF predicts the two helices to form intramolecular contacts. By taking them apart into separate fragments it could be that intramolecular contact sites are now used for interface prediction.

The pair of interactions has a DMI predicted, MOD_GSK3_1 (PAX6 395-402). The peptide PAX6 394-404 was paired with the Pkinase domain but similar to the previous point, it is also put at the beta barrel fold in the N lobe and not the substrate binding site.

run20: PAX6-NGLY1

The PUB domain from Q96IV0, NGLY1, gives good model confidence, >0.8, in binding overlapping disordered fragments of P26367, PAX6. The PUB domain has been solved before alone (2CCQ), the catalytic domain has also been solved bound to RAD23 (2F4M); in the paper that published the PUB domain structure (Allen et al JBC 2006, 10.1074/jbc.M601173200) they also did some mutational analysis to show that there is an interface on the PUB domain that binds the AAA ATPase domain of p97 but the experimental evidence looks not very convincing. Indeed, AF modelled the peptide from PAX6 to bind to an interface adjacent to the one found by Allen et al. There is indeed some hydrophobic pocket and the best 4 predictions comprise that peptide binding to this pocket, however, which hydrophobic residue of the peptide is docked into the pocket varies depending on the length of the peptide; I think that this region in PAX6 could indeed be a linear motif, it is adjacent to the homeobox domain but I don't think that it is part of the homeobox domain.

run21: PAX6-ESRRG

Many short fragments with high model confidence that are scattered over the disordered region. The binding pocket on ESRRG is in the hormone receptor domain and is a known binding pocket for binding to L..LL motifs (ELMDB: LIG_NRBOX).

According to ELMDB, the first and last L go into a hydrophobic pocket and all fragments of PAX6 with high model confidence have more or less two hydrophobic amino acids with three residues in between: PAX6 319-329: DTA**L**TNT**Y**SA, PAX6 203-213: RLQ**L**KRK**L**QR, PAX6 374-384: PPH**M**QTH**M**NS, PAX6 198-208: DEAQ**M**RLQ**L**K, PAX6 128-148: GADG**M**YDK**L**R.

Looking at structures with ESRRG and two different bound peptides: 1KV6 and 1TFC: NCOA1 686-700: RHKI**L**HRL**L**QEGSPS, 2GPO and 2GPP: NRIP1 378-387: SL**L**LHL**L**KSQ, it furthermore became apparent that the hydrophobic residues right before both Leucines are also important for binding since they contact a hydrophobic patch on the other side of the pocket. However, none of the AlphaFold predicted motifs really fit, it is thus questionable whether they can actually bind the pocket.

Structurally speaking, the peptide does not fit that nicely in the hydrophobic pocket. In 2GPO and 2GPV, there is a triad of hydrophobic residues (V/L/I) making contact with the hydrophobic pocket on the domain but here only 2 residues are making contact. Therefore, it seems doubtful to me that this is a motif that can bind to the domain.

run22: PAX6-QRICH1

Difficult to dig deeper because QRICH1 has only one domain (DUF) that binds to C terminus peptide from PAX6. The high confidence peptide is 20 aa long and seems nice with 0.88 model confidence.

The same DUF is also modelled with 0.76 confidence with a very long disordered region (85 aa) that is at the N terminus of PAX6. However, the predicted complex of this disordered region is quite odd, as it has many twists and turns that seem weird to me.

Overall, these predictions look good but it's hard to be very certain about it because nothing is known about the domain in QRICH1 and PAX6 has a long disordered C-terminal region full of S, T, but also some Ps and hydrophobics.

run23: PAX6-KCTD7

The top prediction involves the disordered region of PAX6 (198-208) and BTB_2 domain of KCTD7, with 0.74 model confidence. No trend of increasing confidence when fragments shorten. InterPro describes this domain as one that multimerises for its protein function, e.g. KCTD1 as a transcriptional repressor (3DRX, solves KCTD5 that has a similar fold but shorter in length). Since BTB domain mediates the multimerisation of KCTD, it could be that it requires a certain stoichiometry for binding to its partner. In the HuRI database, KCTD7 was indeed detected to interact with itself. The two highest predicted models put both peptides into the same pocket and both peptides have some sequence similarity albeit from different regions in PAX6. These peptides were also predicted with high model confidence in other runs. Based on the structure 5FTA, BTB domains in their homodimerized form do expose the surface predicted in the top prediction. Therefore, the surface predicted to bind to the peptide would be available. Taken together, the prediction looks plausible.

run24: TTC19-FH

Not inspected because none of the predictions returns model confidence or average interface pLDDT above cutoff (model confidence ≥ 0.7, ordered-disordered prediction with disordered fragment interface plddt ≥ 70; ordered-ordered prediction with intf_avg_plddt ≥ 75).

run25: PEX3-PEX16

PEX3 and PEX16 are two proteins that seem to cooperate to help inserting new peroxisome membrane proteins (PMPs) into the peroxisome membrane. They do so via interaction between PEX3 and PEX19. PEX19 brings the PMPs to the peroxisome where PEX3 and PEX16 sit and mediate then further insertion of the cargo (this is described in review Smith and Aitchison 2013 Nature Rev Mol Cell Biol in Fig 2). However, there is also a study that describes how PEX16 localizes to ER and from there traffics to peroxisomes (Kim et al JCB 2006). The structure 6AJB that has been solved for the interaction between PEX3 and PEX19 was published by Sato et al EMBO J 2010 and describes how an N-terminal SLiM in PEX19 binds to the domain of PEX3. They tried to crystalize the whole protein of PEX3 but only observed residues 52-368. The domain has the exact same fold as predicted by AF. The predicted cytosolic and peroxisomal localization of protein regions and the two TM helices that are shown in Uniprot seem to be wrong for PEX3 according to work cited in Sato et al. They summarize that the N-terminal region of PEX3 contains a targeting signal or anchor for PEX3 to the peroxisomal membrane followed by the domain that is located in the cytosol. No structure has been solved yet for PEX16 but it seems likely that the prediction of two TM helices that are shown in UniProt in this protein is also wrong. AF predicts a globular domain containing the two TM helices and has a nicely exposed loop that carries the putative SLiM that AF predicted to bind to PEX3. It binds onto PEX3 on the opposite side to PEX19 binding, so PEX19 and PEX16 could bind simultaneously to PEX3. Further work on these interactions can be done by submitting the three protein sequences to AF to see what it does. Some other study observed interaction between PEX3 and PEX16 according to Uniprot but the interface really does not seem to have been looked at before nor the interaction studied in detail. All the fragments that contain the putative SLiM in PEX16 are predicted in the exact same way to bind to PEX3; always anchored via a conserved region sitting in PEX16 between residue 160 and 190. Interestingly, the most conserved residues are also those that seem most important for binding. This smells really good.

run26: PEX3-PEX19
This is a positive control interaction since the structure has been solved for this PPI (3AJB) and it is a well known and well studied PPI with an entry for it in the ELM DB: LIG_Pex3_1 (L..LL...L..F). This ELM instance is indeed predicted by AF to be the highest model confidence. Another peptide from PEX19 121-141, FTSCLKETLSGL, scored equally high model confidence. It could be that this other predicted binding site is also true but I believe that it is rather an artefact from AF's insensitivity to mutations.

run27: GABARAPL2-UBA5
The structure 6H8C shows binding of GABARAPL2 domain to LIR motif in UBA5. This motif is not listed on the ELM website for LIG_LIR_Gen_1 because it does not quite fit the regular expression which seems to be defined too narrowly. AlphaFold correctly predicts this interface but only as third highest based on model confidence just hitting the cutoff of 0.7 while using chainB_inf_avg_plddt it scores as fourth best prediction far below the cutoff (67). However, AF recurrently finds peptides including the motif following each other when ranked by model confidence or pLDDT. The top three motifs predicted to bind to GABARAPL2 are not finding the hydrophobic pocket that is filled by a key big hydrophobic residue in the motif and these peptides are also not recurrently predicted. So, I think these are wrong predictions.

run28: GABARAPL2-LZTR1
GABARAPL2 (P60520) has Atg8 domain that is known to bind motifs (LIG_LIR_Gen_1). The domain is modelled with high confidence to bind to different disordered fragments of interacting partner LZTR1 (Q8N653). The second top confident model (when ranked by model confidence) has an aromatic residue tucked into a deep pocket and a branched alipathic residue tucked into another shallow pocket. The top confident model has some kind of increasing trend in model confidence as fragments get shorter, with the shortest one getting the highest confidence. The highest confidence model has a nice increasing model confidence trend but it does not have an aromatic residue fitting into the deep pocket as it is known for LIG_LIR motifs.

Looking at the structure 2LUE, the second top model LZTR1 46-52 GP**F**ET**V**H looks more similar in sidechain positioning compared to 2LUE. Residues highlighted in bold get tucked into the mentioned pockets. This model seems more likely to be true than the best model. However, it also is predicted to bind in reverse order compared to structure 3WIM.

run29: CUL3-KCTD7
Has an ordered-ordered prediction with quite high confidence (0.66) but the contact interface is a tetramerization domain from KCTD7. Therefore it seems unlikely that it is a functional interface.

Two N terminus disordered fragments from KCTD7 with > 0.7 model confidence when paired with the Cullin domain of CUL3. These two fragments are modelled to be binding at the same site of Cullin domain (the site where RING proteins bind to, 1LDJ). In the case of 1LDJ, the RING protein has a long disordered region inserted into the Cullin domain of CUL1, burying a series of hydrophobic residues in the long disordered region. However, the same binding site of the Cullin domain of CUL3 is a bit different, with more surface exposed than CUL1. In this case, the contacts modelled in KCTD7 16-26, with a triple Serine making contact with the Cullin domain, look plausible. The other high confidence peptide KCTD7 1-11, with triple Valine making contact with the Cullin domain, also looks plausible to me.

In the structure of 1LDJ it is really amazing how the partner protein interacts with CUL1 via beta-sheet augmentation but how this extra beta strand becomes part of the integral fold, it is kind of in the middle of the domain. I think AlphaFold feels that there is something missing and is trying to put a peptide there but the overall conformation of the domain is also different at places so that the predicted peptide does not sit at the same position like the one shown in 1LDJ. AlphaFold predicts two different motifs of very different sequence from the N-terminus of KCTD7 to bind there. Given how different the sequences are, this adds another negative point towards questioning the specificity of these predictions.

run30: PNKP-SYP

Top prediction is a disordered fragment from SYP (7-19) paired with the kinase domain of PNKP. The binding surface is different from the nucleotide binding surface (1RC8). This binding interface looks plausible. It was later found that the kinase and phosphatase domain form a structural unit based on published structures. The run is modified to use the kinase and phosphatase domain as an ordered region for prediction with disordered fragments of SYP.

The rerun with a fragment comprising the phosphatase and kinase domain now resulted in one prediction that makes the cutoff. This prediction puts a motif from SYP into the DNA binding pocket of the kinase domain (according to Bernstein et al Mol Cell 2005, 1RC8).

There is another predicting docking a peptide from SYP into the FHA domain of PNKP. It puts it where FHA domains bind their phosphorylated peptides but the SYP peptide has no Ser or Thr.

run31: PNKP-TRIM37

The first prediction involving the combined kinase-phosphatase structure puts a peptide of TRIM37 into the binding pocket where the phosphatase domain would bind single stranded DNA.

Following up is a prediction that involves a disordered region in PNKP binding to the surface of MATH domain of TRIM37 where MATH domain-binding peptides generally bind to. The PNKP peptide differs slightly in sequence from regular expression patterns described for MATH domains in the ELM database. This peptide in PNKP has a known phosphorylation site that stabilizes PNKP protein levels, making the peptide very interesting since this suggests a regulatory role of phosphorylation on the peptide.

There is a second peptide of PNKP predicted to bind to the MATH domain also with high confidence but the sequence is quite different from the first one and very close to the phosphatase domain. There is also a prediction where the FHA domain of PNKP is predicted to bind to a peptide of TRIM37 but the peptide looks very different from known FHA-binding motifs (peptide with phosphorylated threonines), which is of course difficult to predict for AF.

run32: PNKP-XRCC4

XRCC4 and PNKP prediction, there is a peptide from XRCC4 that binds to the phosphatase domain with high confidence. But then I am not sure if this is right because it could be a false prediction of a small peptide easily fitting into the catalytic site of the phosphatase domain. There is a Serine in the peptide, so it is possible that this is where the phosphate group gets cleaved off by the phosphatase domain. After checking more, it is found that XRCC4 is known to bind to PNKP via a phosphorylated motif that binds to the FHA domain in PNKP.

In principle, it would be better to make a rerun where the kinase and phosphatase domain are taken as one fragment since they form 1 structural unit but I think in this case it would not have changed anything. The best prediction put a peptide from XRCC4 into the

pocket of the phosphatase domain where it would bind the single-stranded DNA as seen in 3U7G. Among the first 9 predictions AF put 7 different peptides from XRCC4 into the phosphatase, the others go to the kinase domain. The first prediction that involves the FHA domain of PNKP and contains the FHA-binding motif in the sequence fragment of XRCC4 has a confidence score of 60 and does not put the FHA-binding motif in the pocket but another negatively charged peptide in the sequence (the FHA pocket is very positively charged). The correct prediction where AF puts the FHA-binding motif in the right pocket has a confidence score of 0.58.

run33: TNPO3-GCH1

Top prediction involves the disordered region of GCH1 (16-26) and the superhelical structure of TNPO3, with model confidence 0.71. Since TNPO3 (transportin) is known to transport cargo into the nucleus by releasing the cargo via the competitive binding of GTP-bound Ran (2X19), the peptides from GCH1 are modelled to be at a binding site near where Ran binds in 2X19. It is therefore biologically sound where the peptides are modelled at. The binding site of the peptides from GCH1 is also lined with many arginines, making it very positively charged. The contact modelled by AF in the top prediction looks good, with many charge-charge interactions at the interface. The N terminus of GCH1 has many prolines that are conserved, with three repeats of PAEK or PEAK and two repeats of PPRP.

run34: TNPO3-CAMK2G

Not inspected because none of the predictions returns model confidence or average interface pLDDT above cutoff (model confidence ≥ 0.7, ordered-disordered prediction with disordered fragment interface plddt ≥ 70; ordered-ordered prediction with intf_avg_plddt ≥ 75).

run35: GNAI3-GPSM2

This interaction has been structurally solved (4G5S) and AlphaFold predicted the interface 100% accurately. GPSM2 has multiple GoLoco motifs that AlphaFold predicts individually with high confidence to bind in the pocket on GNAI3.

run36: SYT1-MIP

Both are transmembrane proteins. The top prediction involves the linker between two C2 domains of SYT1 and the MIP domain of MIP. MIP domain is also known as aquaporin domain (transmembrane). However, when the linker is fragmented, it receives lower confidence. I think this is unlikely to be the interaction interface. The linker could be a motif for some other interaction because of its moderately high plddt. There is a structure of a homodimer of SYT1, 2R83, that shows that both C2 domains of one chain are actually interacting with each other and that the linker between both domains interacts with one domain. It is this linker where AF predicts that a peptide would bind to the porin domain of MIP; interestingly, AF predicts the two C2 domains to be independent from each other in the monomeric structure of SYT1, so either AF is wrong or crystallization introduced the packing of both domains against each other but I would rather believe the Xray structure and in this case the peptide would not be accessible to bind to the MIP domain.

run37: FTSJ1-CERT1

FTSJ domain of FTSJ1 is known to bind S-ADENOSYLMETHIONINE (see structure 1EJ0). The top predictions all look very different in that different regions or partially overlapping regions of CERT1 are docked into different sites onto the FTSJ domain. Sometimes the

peptide is docked into the catalytic pocket where the protein methylates adenosines on tRNAs but the peptide is also docked elsewhere. Because of these ambiguities, I believe that the predictions are questionable since they seem to lack specificity but I don't think we can call them definitely wrong.

Another interface was found involving CERT1 368-388, with model confidence 0.70. However, the contacts modelled are mostly backbone-to-backbone. I have previously noticed that AF tends to give higher confidence to complex modelled with secondary structure. So I think this is also a likely false interface.

run38: CAMK2A-SOX5

Kinase domain of CAMK2A with a disordered fragment predicted showed high confidence. The structure predicted by the highest confidence model is weird, with both beta sheet and helix structure.

Kinase domain of CAMK2A is likely serine threonine kinase and in kinase domain prediction, one has to be careful with the two lobes that bind substrate and ATP. It might be interesting to check other high scoring peptide to see if they have S/T that can be phosphorylated and check the crystal structure to find substrate binding pocket. The first two highest scoring peptides do not look convincing because the first one has no S/T in the peptide but it is fit into the catalytic cleft while the second one has positioned the sidechain of a T out of the cleft. The third highest scoring peptide (P35711 131-141) looks nice because it positions the sidechain of an S into the catalytic cleft.

The highest ranking peptides are essentially all over the place from SOX5 and I don't think that AF can predict very well kinase-substrate interactions. Overall, the high-scoring predictions all do not look very convincing.

run39: CAMK2A-CAMK2G

Many high confidence predictions involving different regions in the protein pair. Among them, one ordered-ordered interface gives a really high confidence. The interface is a known DDI in 3did with high zscore. The structure 3SOA only shows one CAMK2A monomer but the publication talks about a dodecamer for which one can download a model from the PDB as well. Looking at this dodecamer and the paper, it becomes clear that downstream of the kinase domain there is another domain referred to as hub domain in the paper which mediates oligomerization, together with the linker between the kinase and hub domain. The best AF prediction for the interaction between CAMK2A and CAMK2G involves both hub domains and is an accurate prediction of the interface seen in the dodecamer.

The second best prediction made by AF involves the hub domain and a bit of the linker sequence from the other partner. Looking at the dodecamer, one can see that where the peptide is predicted to bind on the hub domain is part of the linker sequence bound from the same monomer, so an intra-molecular interaction. So, there is indeed some binding site but not for inter-molecular interaction. Because the linker sequences are different in the structure and canonical uniprot sequence it is very difficult to know which part of the linker is binding on the hub domain and whether this corresponds to the bit of the linker sequence predicted by AF to bind there. In the paper accompanying the 3SOA structure they also investigate how different linker sequences from different isoforms influence Ca-binding site accessibility and thus activation of the complex. There is evidence from 3 other studies that CAMK2G and CAMK2A interact with each other from co-IP experiments but these were large-scale studies. It is likely that no one has studied the interface between CAMK2G and CAMK2A and thus would be something new.

run40: ACTB-ACTG1

Two actin proteins are predicted to have high confidence DDI. The interface itself that is predicted by AlphaFold looks very interesting, it indeed looks like a polymerization interface because both domains interact with opposite sites. interactome3D would model this interaction with the structure 4JHD as a template but this one looks quite different, it's not the same interface and needs according to the authors a third protein for polymerization. Digging deeper in PDB for structures of ACTB, I found structure 6ANU which shows the same interface that AF predicted between ACTB and ACTG1, so the interface is probably right.

This is also a very interesting case. Based on the review by Vedula and Kashina (J of Cell Signal 2018, 10.1242/jcs.215509), it is still an open question whether the different actin forms that exist in human can form heteropolymers or not. Some studies find this in vitro, other find intermingled homopolymers of beta and gamma actin. Both actins co-occur in many cell types while alpha-actin is more specifically expressed in muscle. It seems really tricky to solve this since actins are highly studied and actins are also super similar in their sequence, so it could be that in a somewhat artificial system, beta and gamma actin can interact because the interface residues are identical but in vivo they would rather not interact and rather form homopolymers. In the end, whether ACTB and ACTG1 indeed interact in vivo is the only open question.

run41: RARB-PSMC5

PSMC5 has been repeatedly modelled by AF to have a high confidence peptide that binds to partners with Hormone_recep domain. The peptide is 132-141 DP**L**VS**LM**MVE. Residues highlighted in bold are the ones tucked into the hydrophobic pocket. However, this peptide does not match with the consensus of LIG_NRBox (^PL..LL^P), especially in this peptide P precedes the first L. I am not sure why P is disallowed at first position as ELM has not described much about the sequence composition of the motif. I think it might be too early to reject this peptide because the highlighted residues are indeed hydrophobic and can serve similar functions as those in the regex.

I looked at the HuRI network of PSMC5 too, and found that the interactors seem to be enriched with the Hormone_recep domain, making this interface even more plausible.

run42: DCX-BICD2

DCX has two DCX domains and all good predictions involve the N terminus DCX domain. The N terminus DCX domain is known to bind Tubulin. AF modelled a different interface on the N terminus DCX domain to bind to disordered fragments from BICD2.

The DCX domains have a C-terminal part that is not confidently predicted by AF to be part of the fold. When excluding this part from the first DCX domain, AF models peptides to bind to the area where this last part is predicted to be located in the monomeric structure from AF. When we use a DCX domain that contains this last bit, then AF predicts other peptides from BICD2 to bind on the opposite side of DCX. There is no consistency in these predictions.

There are no other predictions between ordered-ordered or disordered fragments binding to ordered domains in BICD2 that make the cutoffs. BICD2 however, also only consists of large helices. Nonetheless, it could be that both DCX domains together bind to one of these coiled coil helices in BICD2.

run43: DCX-ZBTB10

A possible prediction involves the first DCX domain of DCX and a peptide of ZBTB10 261-271. This prediction is not influenced by the actual domain boundaries because the peptide is not docked into the pocket where a region a little C-terminal of the domain might bind to. This is the case for the second best prediction involving the first DCX domain and peptide 604-614. According to chainB inf avg plddt these are the only two prediction that make the cutoff when looking at chainB as a disordered region. ZBTB10 has a lot of disorder and probably many motifs. DCX has two DCX domains and a bit of disorder. Looking into available PDB structures then the DCX domains are known to bind to microtubules. There is one structure with the first DCX domain bound to microtubules (6RFD). It seems though that the pocket where ZBTB10 261-271 is predicted to bind is not occupied in this complex. AF does not predict slightly extended versions of this peptide with reasonable confidence to bind to this pocket.

A peptide was also predicted to bind in beta-sheet augmentation to the last beta strand of the BTB domain with reasonable model confidence and chainA_intf_avg_plddt scores but the ZBTB10 model might have its own beta strand C-terminal of the current domain boundaries that AF predicted to complement the last beta strand of the domain as predicted in the full length model of ZBTB10.

AF also predicts a contact between the ZnF domain of ZBTB10 and the first DCX domain but it does not look very likely and I think the ZnF fold is perturbed.

run44: PSMC5-ESRRG

The interaction has quite some high confidence predictions. The highest scoring peptide is P62195, PSMC5, 132-141, DP**L**VS**LM**MVE. The three hydrophobic residues make nice contacts with the hydrophobic pocket and surface of the domain. Another disordered fragment from PSMC5 binding to the same domain, IKKLWK, also looks promising. However, there is some possibility that these are artefacts because AF is not very specific when it comes to detecting single mutation in known motifs. The sequence alignments are not helpful unfortunately because the whole PSMC5 is super conserved.

Nonetheless, interaction between PSMC5 and ESRRG looks promising because the alternative name is thyroid hormone receptor-interacting protein 1, TRIP1.

run45: PSMC5-RORB

The highest confidence prediction involves a disordered fragment from PSMC5 and it is the same as run44. The ordered region from RORB is the same domain, hormone receptor domain, as run44.

It is interesting to see AF predicting similar DMI with high confidence from two different proteins. Same observation as run44.

run46: WAC-NFE2L2

WAC and NFE2L2 are largely disordered. WAC has a WW domain. AF predicts recurrently a sequence close to the N-terminus of NFE2L2 to bind to the WW domain that are known to bind proline-rich motifs. The putative motif in NFE2L2 does not contain prolines and is not docked onto the WW domain in any way like other WW domains, e.g. 1EG4. These are likely wrong predictions. While the motif interface pLDDT is reasonably high for these predictions, the model confidence does not reach the 0.6. There are no other predictions that make the cutoff.

run47: WAC-MOBP

Not inspected because none of the predictions returns model confidence or average interface pLDDT above cutoff (model confidence ≥ 0.7, ordered-disordered prediction with disordered fragment interface plddt ≥ 70; ordered-ordered prediction with intf_avg_plddt ≥ 75).

run48: STX1B-FBXO28

The top model has 0.76 model confidence that utilizes the disordered region 1-22 of STX1B and Fbox + helix bundle domain (63-221) of FBXO28. The interface involves the disordered region of STX1B forming a 310 helix structure with the helices from the Fbox domain. Note that the Fbox domain annotated by InterPro is from 61-109, while the ordered region that I used for prediction is 63-221. The Fbox domain is known to mediate PPI but it is not used by AF to model the interaction in this prediction. Region 1-22 of STX1B is conserved only in recent homologs. The plddt of the disordered region is low, <60 for all residues.

The second top model has 0.75 model confidence that involves the syntaxin domain (23-237) of STX1B and disordered region 354-368 of FBXO28. The disordered region of FBXO28 is at the C terminus and conserved. However, the plddt of the peptide is low and adopts a 310 helix kind of structure. A slightly different prediction involving fragments of the proteins (27-219 STX1B and 345-363 FBXO28) returned 0.73 model confidence. The peptide adopts a helical structure but is placed on a different surface of the syntaxin domain. Although the peptide 345-363 has good plddt (mosty >60), I am not sure if this is the right interface. One prediction pairs the full length of STX1B with the disordered region 354-368 of FBXO28 and returned 0.71 model confidence. The interface is similar to that of the syntaxin domain (23-237) of STX1B and disordered region 354-368 of FBXO28 with low plddt. This region 354-368 in FBXO28 could be an nuclear localization signal (NLS), where ELMDB also predicts quite a few NLS, and therefore unlikely to be the interface for the interaction.

Next top prediction has 0.749 model confidence that involves the C terminus of the syntaxin domain (220-232) of STX1B as disordered region and the Fbox + helices domain of FBXO28 (63-221). The interface is formed by the peptide adopting a helical structure with the Fbox + helices domain. The plddt of the peptide is good, with all residues above 60 plddt. Nonetheless, another prediction involving slightly longer peptide from the same region of STX1B has a much lower model confidence (0.55). The interface modelled is not exactly the same as it is a little bit shifted. Unsure if this is a good interface.

I tried to find more molecular studies on the two proteins but I can’t find much. STX1B is known to function in docking of synaptic vesicles at presynaptic active zones while FBXO28 probably recognizes and binds to some phosphorylated proteins and promotes their ubiquitination and degradation. Weirdly, STX1B is known to localize to membrane while FBXO28 has not much information on subcellular localization but studies have shown that it interacts with topoisomerase using its Fbox domain (the bundled helices are not needed for interaction). Out of all the predictions, I think STX1B 27-219 + FBXO28 345-363 and STX1B 220-232 + FBXO28 63-221 are most likely to be the interface, as their peptides are modelled with good plddt and both achieved model confidence higher than 0.7.

run49: STX1B-MMGT1

Top prediction involves the Syntaxin domain of STX1B and the disordered region of MMGT1 (23-31) with confidence 0.73. A slightly longer fragment has a slightly lower confidence but looking at the structure, the two peptides have different angles to the Syntaxin helical bundle. Since the interfaces modelled by AF differ a lot despite using the same peptide and its extended counterpart, the modelled interfaces do not look genuine.

run50: STX1B-VAMP2

Interactome3D models an interface between both proteins based on the structure 3HD7/3IDP where STX1A interacts with VAMP2. STX1A and STX1B are very similar in structure.

STX1B is predicted in closed conformation, which we know because structures exist of STX1A bound to Munc18 where it is in this closed conformation with the long C-terminal helix comprising the SNARE domain folding back onto the syntaxin domain. However, when bound to VAMP2 we can see the open conformation where the long helix is made available to bind in coiled-coil like manner to VAMP2 and SNAP25 helices.

Based on this available structural information we designed different fragments of the extended SNARE domain of variable length. VAMP2 is a short protein of 116 residues consisting of a long helix and about 30 disordered residues at the N-terminus. The most confident predictions obtained for these fragments is the one modeling a coiled-coil interaction between the extended SNARE domain and the helix of VAMP2 but the model confidence is slightly below the cutoff. Predictions with the disordered N-terminal region of VAMP2 remain far below cutoffs.

run51: CSNK2A1-CSNK2B

Nice prediction with overlapping fragments showing increasing model confidence. This interface has been solved before in two structures: 4DGL and 6Q38; prediction is highly accurate, and is probably a DMI that is not in ELM yet.

run52: EBF3-EBF2

Dimerization of the EBF family already known and solved (3MUJ). AF predicts the middle domain of both proteins called TIG as the dimerization interface as top prediction but in head to tail orientation while the structure 3MUJ shows head to head orientation. Followed closely up in terms of score (avg_intf_plddt) is the fragment comprising the TIG domain and the helix loop helix domain which are predicted accurately as seen in the structure.

The third best prediction involves the N-terminal DNA binding domain as the dimerization interface. Does not look so convincing to me but still got a very high score. The fourth best prediction is the helix-loop-helix domain alone as dimerization interface, still with a score of 90. There are more predictions that make the cutoff that involve various disordered regions of either protein and ordered fragments from the other involving interfaces used for dimerization but I guess that these predictions are likely wrong.

run53: PEX12-TREX1

The disordered region of PEX12 215-312 (98 residues long) is predicted with high confidence. One fragment of it achieved even higher confidence but when this fragment is further fragmentate, their confidence is not as high anymore. After checking the protein on InterPro, this domain is the exonuclease domain of TREX1 that binds to ssDNA (2OA8). In this crystal structure, it shows the pocket modelled by AF to bind PEX12 215-312 is bound to a ssDNA, with the phosphodiester bond of ssDNA making interactions with the backbone of the domain chain and some hydrophobic side chain (leucine) making hydrophobic interaction with the base of the nucleotide. Interestingly, AF seems to have memorized this crystal structure because the bound ssDNA has a curved structure and AF also models the long disordered region to have an odd curve. I think this interface is unlikely to be true because the bound magnesium ions coordinate with the oxygen in the phosphodiester bond of ssDNA and the modelled helix places hydrophobic sidechains to the cavity where magnesium ions bind.

A very short fragment of PEX12 12-16 at the N terminus is modelled with high confidence with a very negatively charged pocket in the domain of TREX1. It is unusual to have a peptide binding pocket with such a high negative charge. Further checking revealed that this domain binds magnesium ion and nucleotides. The short fragment fits into the magnesium binding pocket and thus this is unlikely to be true.

run54: PRKAR1A-PRKAR1B

Best model is an ordered-ordered prediction with 0.83 confidence. It is a homo-DDI (RIIa domain) dimerization and has been solved in 2EZW.

An additional disordered fragment (PRKAR1A 360-372) predicted with high model confidence but low pLDDT with the cyclic nucleotide binding domain of PRKAR1B. Referencing available structure of cNMP binding domain (1NE4), there are two beta barrel folds in the domain that bind to cyclic nucleotides. AF fits the disordered fragment on a hydrophobic surface near the beta barrel but not in the cNMP binding pocket. Although this could be another binding site, the binding makes little sense to me because the disordered fragment is at the C terminus of cNMP binding domain of PRKAR1A, meaning that the sequence would have to loop back to make this contact. In the previous bullet point, it seems very likely that the dimerization of the two proteins are mediated by the RIIa domain (N terminus), so it seems not so plausible to me that at the C terminus they make contact again. This is likely a false positive interface.

run55: ASF1A-H4C8

The interaction between both proteins has been solved (5C3I). However, this structure shows that the motif in H4 sits at the very C-terminus and binds in beta sheet augmentation to ASF1A in the same pocket like AF predicted but using an N-terminal peptide of H4. I think the problem is that the C-terminal region of H4 was made part of the domain of H4, which I agree was hard to see from looking at the monomeric AF structure for full length H4; I checked further down in the predicted structures but the first ordered-ordered prediction has a model confidence of 0.25 and does not find this mode of binding either. One could rerun this by taking the C-terminal peptide of H4 as disordered region just to see whether AF would then get it right but in principle this is a false positive prediction; the N-terminal peptide also shares no sequence similarity with the C-terminal motif.

run56: RARS1-CCDC115

There is only one prediction that makes the cutoff for model confidence or/and motif pLDDT. This prediction involves RARS1 1-21 as a disordered fragment that is modelled to bind as a helix to the two helix coiled-coil domain of CCDC115. A shorter fragment of the motif is placed elsewhere. The helix of CCDC115 to which the peptide is predicted to bind has more hydrophobic residues along the helix on that side so I would think that a longer partner chain would be able to bind there. Thus, this interface does not seem likely to be true.

run57: UBE3A-TAT

Not inspected because none of the predictions returns model confidence or average interface pLDDT above cutoff (model confidence ≥ 0.7, ordered-disordered prediction with disordered fragment interface plddt ≥ 70; ordered-ordered prediction with intf_avg_plddt ≥ 75).

run58: VAMP4-MFF

Top prediction is two ordered regions that are both helical. Both proteins have only helical regions and the rest are disordered. Interestingly, despite the top predicted interface having only 0.71 model confidence, both chains have very high plddt for their residues at the interface (95 for VAMP4 and 90 for MFF). Because of their high plddt, it could be a genuine interface. The helix in VAMP4 definitely has an interface there because one side is rather hydrophobic while the other side is rather hydrophilic. MFF could bind there with its helix or via another helix that it has. The binding does not show that many nice contacts, i.e. some hydrophobic residues on the VAMP4 helix still remain exposed.

run59: PEX16-MMGT1

Not inspected because none of the predictions returns model confidence or average interface pLDDT above cutoff (model confidence ≥ 0.7, ordered-disordered prediction with disordered fragment interface plddt ≥ 70; ordered-ordered prediction with intf_avg_plddt ≥ 75).

run60: PLP1-SLC16A2

Not inspected because none of the predictions returns model confidence or average interface pLDDT above cutoff (model confidence ≥ 0.7, ordered-disordered prediction with disordered fragment interface plddt ≥ 70; ordered-ordered prediction with intf_avg_plddt ≥ 75).

run62: SNRPB-GIGYF1

GIGYF1 is a very long protein with many disordered regions. It has a GYF domain that is known to bind proline-rich sequences. SNRPB has many proline-rich sequences in its C terminus. Some proline-rich motifs are predicted with high pLDDT to bind the GYF domain (these are the top predictions).

Another highly ranked prediction involves the LSM domain of SNRPB with various disordered fragments from GIGYF1. However, checking InterPro entry as well as structures showing LSM domain, it seems like LSM domain is predominantly involved in multimerization with other SNRP proteins to form the SMN complex involved in splicing (1H64). Therefore, the models involving this domain with disordered fragments look unlikely to be true to me.

Digging deeper into the top predictions, comparing the binding modelled by AF between SNRPB 231-240 and GYF domain of GIGYF1 with 1L2Z, the peptide is oriented differently. However, from 3FMA, one can see different ways a peptide binds to the same surface of GYF. In 3FMA chain E and P show a similar way of binding to that modelled by AF. The peptide sequence in 3FMA is also different from 1L2Z, but importantly, there are three prolines in the peptide that always orient the same to the hydrophobic surface formed by the GYF motif on the GYF domain. This orientation of the 3 prolines is captured by AF.

AlphaFold repeatedly predicts the PPGM motif in the same pocket. This motif occurs multiple times in the C-ter tail of SNRPB. On the ELM website, the LIG_GYF motif is described to bind proline-rich sequences and they also cite the structure 1L2Z but they say that flanking positively charged residues seem to be important for binding to the GYF domain. Indeed, in the crystal structure there are some negatively charged residues on the GYF domain. Interestingly, the GYF domain from GIGYF1 does not or only partially has those. It also differs in that it has a deeper hydrophobic pocket which is filled with a Trp in the crystal structure. So, it could well be that the GYF domain from GIGYF1 binds somewhat different proline-rich peptides. The interaction between GIGYF1 and SNRPB has not been described before other than in HuRI. Functionally, it would be probably a new connection because GIGYF1 is not known to function in splicing as far as I can see and thought to be localized to the cytoplasm. GIGYF1 however, has also interacted with SNRPA and SNRPC in HuRI. They also have 1 or

some more occurrences of the PPGM motif. If this mode of binding is true then it would be somewhat of a new mode of binding or in the most conservative case an extension of the known binding mode of LIG_GYF.

Alignment of 1L2Z chain A (GYF domain) with the GYF domain from GIGYF1 (476-535) shows that the sequences are not very conserved. Structural superimposition of the two GYF domains reveal that the overall fold is conserved, including the majority of the binding pocket except for the hydrophobic pocket filled with a W. The peptides of the two structures have their PPPG in similar orientation. Following this sequence is a M from SNRPB that is tucked into the hydrophobic pocket and H for 1L2Z that is exposed to the environment. The sequence that follows is R for both, with the one in SNRPB exposed to the environment and possibly forming a hydrogen bond with the Q on the domain, and that in CD2 (1L2Z) forming salt bridge with an E from the domain.

Later a structure of the GYF domain of GIGYF1 was published binding to a similar motif found in TNRC6 further supporting the correctness of these predictions.

run63: ARHGEF9-VEZF1

Top prediction has 0.74 model confidence with the fragment from VEZF1 (375-385) making contact with the RhoGEF domain of ARHGEF9. The top predictions all put the peptide at the same binding site of the RhoGEF domain. In terms of conservation, all the peptides from VEZF1 are well conserved. Nonetheless, the prediction looks like a very questionable one, at least it seems like the predictions do not make use of the GTP/GDP binding pocket for which I did not find a structure that shows where it precisely is located but based on an abstract of an article and InterPro entries it seems to be between both structural entities that form one larger domain, the GEF domain and the PH domain (IPR000219). There is absolutely no consistency in the two peptides from VEZF1 selected to bind to the same surface on the GEF domain of ARHGEF9; VEZF1 also seems to be of very weird type, AF has a hard time to make sense out of this protein.

run64: MIP-MFF

Not inspected because none of the predictions returns model confidence or average interface pLDDT above cutoff (model confidence ≥ 0.7, ordered-disordered prediction with disordered fragment interface plddt ≥ 70; ordered-ordered prediction with intf_avg_plddt ≥ 75).

run65: VEZF1-PRKAR1B

Not inspected because none of the predictions returns model confidence or average interface pLDDT above cutoff (model confidence ≥ 0.7, ordered-disordered prediction with disordered fragment interface plddt ≥ 70; ordered-ordered prediction with intf_avg_plddt ≥ 75).

run66: VEZF1-KCTD7

Top prediction involves the disordered region of VEZF1 (360-380) and the BTB domain of KCTD7. The disordered region overlaps with the top prediction in run63 that models the interface between VEZF1 (375-385) and the RhoGEF domain of ARHGEF9. Despite AF modelling a 310 helix structure in the disordered region of VEZF1 (360-380), the contacts modelled at the interface do not look very convincing. It could be that the disordered region (360-385) is a functional motif for other interactions and AF detects that and tries to fit it into the domain. It could also be that, to form the binding interface, it needs multiple copies of BTB domain, which is not used in this prediction. The VEZF1 peptide is put in the same pocket like

the PAX6 peptides from run23 but the sequences look different, it is however the same peptide in VEZF1 like in the prediction with ARHGEF9.

run67: APTX-FLAD1

Has overlapping fragments with increasing confidence: APTX, N terminus disordered region 5-12 and 6-13, paired with MoCF_biosynth or a domain of unknown type (not matched to a Pfam or SMART domain) that is between the MoCF and PAPS_reduct domain of FLAD1. It also predicts the same N-terminal region of APTX into the PAPS_reduct domain. The disordered fragments from the region 8-15 of APTX showed high confidence model confidence but below the cutoff pLDDT score when modelled with the PAPS_reduct domain of FLAD1. Checking the structure of PAPS_reduct domain in complex with adenosine phosphosulfate shows that the peptide is modelled by AF to be in the binding pocket of adenosine phosphosulfate. This is likely a false prediction.

For the N-terminal part of APTX AF is quite confident when it models it into the MoCF domain or the other unknown domain of FLAD1. There are multiple predictions with different overlapping fragments that make the cutoff. However, AF is more confident with both metrics when the peptide is modelled into the MoCF domain. This domain has a pretty substantial pocket that is actually in the monomeric structure of FLAD1 occupied by another region of FLAD1 with low pLDDT. However, when APTX 10-15 is used for modelling, the orientation of the peptide is reversed. MoCF_biosynth domain is known to trimerize for its activity and is known to bind molybdopterin. MoCF_biosynth binds molybdopterin on a site close to where AF models the peptide to be (refer to 1DI6, https://doi.org/10.1074/jbc.275.3.1814 that solves the structure of a bacterial protein with the same domain. They mentioned 49D and 82D to be important for catalytic activity)

APTX with the unknown domain of FLAD1 does not reach the model confidence cutoff, only the motif pLDDT cutoff. It puts the same peptide as beta-sheet augmentation to the domain while in the predictions for the MoCF domain, the peptide is put in helical conformation.

The only predictions where disordered regions in FLAD1 are predicted to bind to folded regions in APTX involve the FHA domain of APTX and correspond to two completely different disordered regions in FLAD1.

run68: FBXO28-PSMC3

Top prediction is coiled-coil interaction between regions from the two proteins that are modelled by AF monomer as long helices. The plddt of all residues are very high. This interaction looks convincing. The only problem is that one helix is shorter than the other, while for a common coiled coil interaction, both helices are usually equally long.

The second best prediction based on model confidence involves a disordered region from FBXO28 (51-61). The modelled complex does not look convincing because the peptide is quite hydrophobic and the residues do not make much contact with the domain. The peptide is predicted to bind to the first domain of PSMC3 which as far as I was able to find, does not have catalytic activity.

There are only these two predictions that make the cutoff for model confidence, none make the cutoff when looking for disordered regions in PSMC3 predicted to bind to FBXO28. The other way round there is the peptide mentioned above and a C-terminal disordered region of FBXO28 predicted to bind to the same first domain in PSMC3 but predicted to bind to a different side. The C-terminus of FBXO28 is very charged, maybe a localization signal. Both motifs in FBXO28 are somewhat recurrently predicted to bind to the domain in PSMC3.

run69: CAMK2G-ESRRG

Many high confidence predictions in a disordered region of CAMK2G. The whole disordered region used as a fragment for prediction also returned high confidence (0.78). In this long disordered region, AF puts the third highest model confidence peptide in the domain pocket. The top three highest confidences are very similar in terms of confidence. The motif detected by AF resembles LIG_NRBOX with the motif L..LL. CAMK2G 300-310: LKGAI**L**TT**ML**V -> looks plausible to me because the M is hydrophobic and it is possible to substitute for the role of L in the regex. CAMK2G 315-325: SA**A**KS**LL**NKKS -> Also possible but the A is fitted into a quite deep hydrophobic pocket where known structure (refer to run21) shows that it is L that gets fit into the pocket. A might have too short of a hydrophobic side chain to make a good contact with the deep pocket. CAMK2G 355-365: QEPAP**L**QTAME -> not so good IMO because the hydrophobic contact is less extensive as the peptide found above. Another interesting observation: CAMK2G 285-423 (139 aa) prediction resulted in 0.78 model confidence, which is very high for a disordered region that long. In this case, **CAMK2G 300-310** is fitted into the hydrophobic pocket, adding weight to the fact that this could be the correct peptide. This reminds me of the extension analysis with DMI where extension of motif can improve prediction results.

A pairing of ordered-ordered region prediction returned high confidence (0.83). This involves Zn finger from ESRRG and CaMKII association domain at the C terminus of CAMK2G. The binding is close to but not in the Zn binding pocket, which is good. CaMKII association domain of CAMK2 has been shown to oligomerize with other CAMK2 in 1HKX.

Looking at the monomeric structure of ESRRG and CAMK2G, it looks possible that the C terminus association domain of CAMK2G to bind to ESRRG via Zn finger domain of ESRRG and the hormone receptor domain of ESRRG binds to the long and disordered region separating the two domains found in CAMK2G. This makes a multi-site binding between two proteins and a very interesting case.

run70: XRCC4-LIG4

The structure for this interaction has been solved: 3II6 and 1IK9. Looking at the structure of 3II6, the two proteins interact with each other via XRCC4 first forming a homodimer with its coiled-coil domain, then around the homodimer binds the tandem BRCT domains of LIG4. The BRCT domains are separated by a structurally less defined region that most likely forms two helices upon binding to XRCC4. Not sure if this can be seen as domain-motif or domain-domain interaction, probably something in between. It is not so clear from the monomeric AF model of full length LIG4 that both BRCT domains form a functional unit but I guess one could have also made a fragment comprising both domains and the linker sequence. Runs so far were made with both BRCT domains individually and the linker sequence individually and further rerun has to be done by using the BRCT domain tandem as one structural unit.

The top prediction involves a motif at the C-terminus of XRCC4 that is predicted to bind to the last BRCT domain of LIG4. I think the prediction is wrong because of the solved structure. The prediction also does not look like how other motifs bind to BRCT, i.e. the protein FANCJ (LIG_BRCT_BRCA_1). However, the C-terminus of XRCC4 certainly carries one or two motifs. One is annotated in Proviz as WD40 domain binding. The very C-terminus is a class 3 PDZ-binding motif. The whole region is very conserved. Maybe this is why AF tries to put peptides from this C-terminus in various domains, including the DNA ligase domain of LIG4 (fourth top prediction). So, the top two predictions involve this C-terminus and reach high confidences in both metrics (model confidence and intf_avg_plddt).

The third highest prediction involves the XRCC4 N-terminal domain plus one long helix (taken as one ordered region) and the 2nd BRCT domain. This interface is exactly the same interface that is seen in the structure 3II6 where part of the BRCT domain also contacts the XRCC4 helix.

The 6th best prediction involves the linker between both BRCT domains and the XRCC4 helix. Despite the fact that XRCC4 is in monomeric form in our prediction and that the BRCT domains are missing, AF correctly models the contacts between the linker and the single XRCC4 domain as they can be seen in the structure 3II6. This model meets both cutoffs, for model confidence and pLDDT.

Rerun using the BRCT domain tandem as one structural unit completed. The tandem BRCT fragment ranks 7th with the coiled coil XRCC4 fragment based on model confidence and second for ordered-ordered fragment pairs when ranked by avg interface plddt. The prediction that is still ranked first is the single BRCT domain binding to the coiled coil fragment (92 vs 89 avg intf plddt score).

run71: TMEM237-MFF

Not inspected because none of the predictions returns model confidence or average interface pLDDT above cutoff (model confidence ≥ 0.7, ordered-disordered prediction with disordered fragment interface plddt ≥ 70; ordered-ordered prediction with intf_avg_plddt ≥ 75).

run72: HNRNPK-TH

In the full length structural model of HNRNPK the first 2 KH domains are predicted to pack against each other using an interface that is also predicted to bind to the TH peptide 61-71. This region indeed overlaps with a Pfam HMM that seems to find some pattern in this disordered region but nothing is known about this “structural”(?) motif. It predicts 3 occurrences of it in the N-terminal region of TH but the third one is the most conserved and this is the one predicted to bind to the second KH domain. Two other motifs overlapping with 61-71 are also predicted to bind to this KH domain. The residues that are part of all three motifs are predicted to bind to the KH domain in the same way. One prediction below the model confidence cutoff predicts the motif to bind to the third KH domain but in a different way.

run73: OTX2-RPS26

Not inspected because none of the predictions returns model confidence or average interface pLDDT above cutoff (model confidence ≥ 0.7, ordered-disordered prediction with disordered fragment interface plddt ≥ 70; ordered-ordered prediction with intf_avg_plddt ≥ 75).

run74: MFF-MMGT1

Not inspected because none of the predictions returns model confidence or average interface pLDDT above cutoff (model confidence ≥ 0.7, ordered-disordered prediction with disordered fragment interface plddt ≥ 70; ordered-ordered prediction with intf_avg_plddt ≥ 75).

run75: PUF60-TH

The top prediction involves using both RRM domains of PUF60 as one ordered region and a disordered polyA peptide from TH. The peptide is put at the same position where the Nbox would bind as shown in the NMR structure 2KXH. However, the predicted peptide has some different sequence: solved structure: LxxAxxI, model: VxxAxxV, and there are no recurrent predictions. Another prediction involves the third RRM domain of PUF60 and another peptide in TH which tugs a Trp in a pocket but it does not look very convincing.

Prediction involving disordered fragments from PUF60 and ordered region (Biopterin_H domain) from TH returned a maximum of 0.78 model confidence. This is likely false interface because the short peptide is fit into the biopterin and iron binding pocket of the enzymatic domain (refer to run72 for example). The second best prediction is also fitted at the same site, therefore also likely a false interface.

Interestingly, the disordered region of PUF60 302-461 is modelled with 0.69 model confidence with the Biopterin_H domain of TH. The long disordered region makes contacts with two regions of the domain, one at the iron binding site (likely false) and another coiled-coil interaction at the C terminal helix of Biopterin_H domain. This coiled-coil interaction is repeated in a shorter disordered fragment of PUF60 (317-347, third best prediction (0.77), the same C terminal helix in the long disordered region). This coiled-coil interaction looks like a plausible interface.

I tried finding more information about this ACT-like domain but to no avail. InterPro says that it homo-dimerizes using the beta strands like in 1Q5V, but the fold is not exactly the same. The ACT-like domain in TH is special in the way that the last beta strand is formed by its N and C termini by looping back to meet each other. I cannot find much information about this domain.

run76: PUF60-QRICH1

One long disordered region of PUF60 (1-128) is modelled with high model confidence with DUF of QRICH1. In this region, 111-121 is modelled at the interface. This region when fragmented from the long disordered region also showed high confidence (0.86). This fragment tucks a R into a very deep negatively charged pocket but the rest of the peptide seems to make questionable contact with the DUF domain.

Top prediction with ordered region in QRICH1 and peptides in PUF60 either put the linker helix between the first two RRM domains or the N-terminal long helix in PUF60 or another helical peptide at 442-461 at two different places on the DUF domain. I think that the helical linker between both RRM domains is not accessible for this mode of binding because the key residues are making intramolecular contacts to the RRM domains in the AF monomer PUF60 model.

3 different peptides are predicted to bind to the tandem PUF60 domain. In principle, the long disordered N-terminal region of QRICH1 is full of potential helical peptides of pattern hydrophobic-x-x-Ala-x-x-hydrophobic, which is the kind of peptide that is like the Nbox motif that can bind to PUF60 and the three different peptides are also predicted to bind to the same pocket.

There are also 4 different peptides in QRICH1 predicted to bind to the third RRM domain.

run77: MAB21L2-AP1S2

The top prediction involves Clat_adaptor_s domain of AP1S2 with the disordered fragment (215-220) of MAB21L2 (78 motif pLDDT, 0.77 model confidence). The motif is predicted recurrently with variable length but the disordered region is generally very short because it is a loop within the domain of MAB21L2. AF also made a disulfide bridge between motif and domain. Not sure this is correct. Looking at the structure 1W63 that shows the large Ap1 clathrin adaptor core complex where there is a fold similar to the one in AP1S2, one can see that the region where the peptide is predicted to bind would in principle be accessible for binding. This domain Clat_adaptor_s is known to bind motifs from ELMDB but no structure has been solved in terms of this domain and its bound peptide. The disordered fragments from

the previous point also do not match with any ELM class that binds to Clat_adaptor_s. Other good predictions use the Mab-21 domain of MAB21L2. Two overlapping disordered fragments (146-154, 0.68 and 153-157, 0.75) had good confidence with the domain but they are modelled to be at different binding sites, so it does not look likely to me that this is the binding region.

run78: PRKAR1B-QRICH1
The motif in PRKAR1B is at the very C-terminus of the protein and also matches a PDZ-binding motif. There is only one prediction that makes the model confidence cutoff but it does not meet the pLDDT cutoff. The C-terminal peptide of PRKAR1B binds to the only domain of QRICH1 but extended or smaller versions of the motif are only predicted with very low score then to bind to the domain so no recurrence here. The prediction therefore looks unlikely to be functional. No other predictions make the pLDDT cutoff.

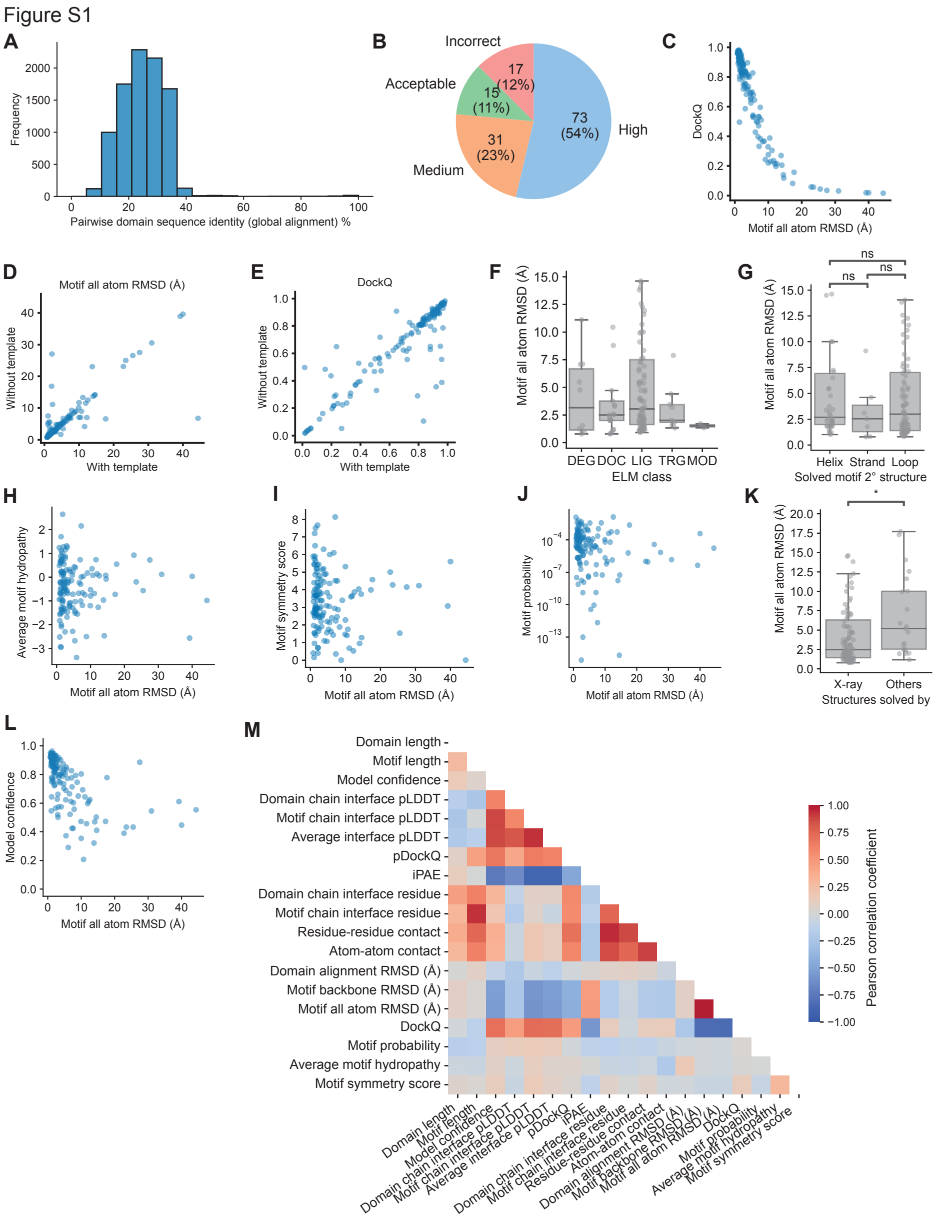

Appendix Figure S1. Benchmarking of AF on DMI interfaces using minimal interacting regions.

A Pairwise sequence identity of domains in the DMI positive reference dataset. **B** Proportion of high, medium, acceptable and incorrect models predicted by AF from the positive reference dataset as classified by the DockQ score. **C** Scatterplot of DockQ vs motif RMSD for DMIs from positive benchmark dataset. Pearson r = -0.85, p-value < 0.0001. **D-E** Motif RMSD and DockQ scores of structures for DMIs from positive benchmark dataset predicted by AF with and without the use of templates. Motif RMSD: Pearson r = 0.81, p-value < 0.0001. DockQ: Pearson r = 0.88, p-value < 0.0001. **F** Accuracy of AF DMI predictions stratified according to the annotated functional categories of DMIs in the ELM DB. DEG=degron, DOC=docking, LIG=ligand, TRG=targeting, MOD=modification. **G** Accuracy of AF DMI predictions stratified according to the secondary structure element formed by the motif in the solved structure. **H-J** Scatterplot of various motif features vs motif RMSD determined for models and structures of DMIs from positive benchmark dataset: H motif hydropathy, Pearson r = -0.03, p-value = 0.72, I motif symmetry, Pearson r = -0.08, p-value

= 0.38, J motif regular expression degeneracy, Pearson r = -0.04, p-value = 0.66. **K** Accuracy of AF DMI predictions stratified according to the method used to solve the structures in the benchmark dataset, Mann-Whitney-Wilcoxon test two-sided p-value = 0.017 test statistics = 811 **L** Scatterplot of model confidence of predicted models vs motif RMSD determined from superimposing the predicted models with structures of DMIs from the positive benchmark dataset. Pearson r = -0.55, p-value < 0.0001. **M** Correlation matrix of different prediction variables and prediction outcomes.

Figure S2

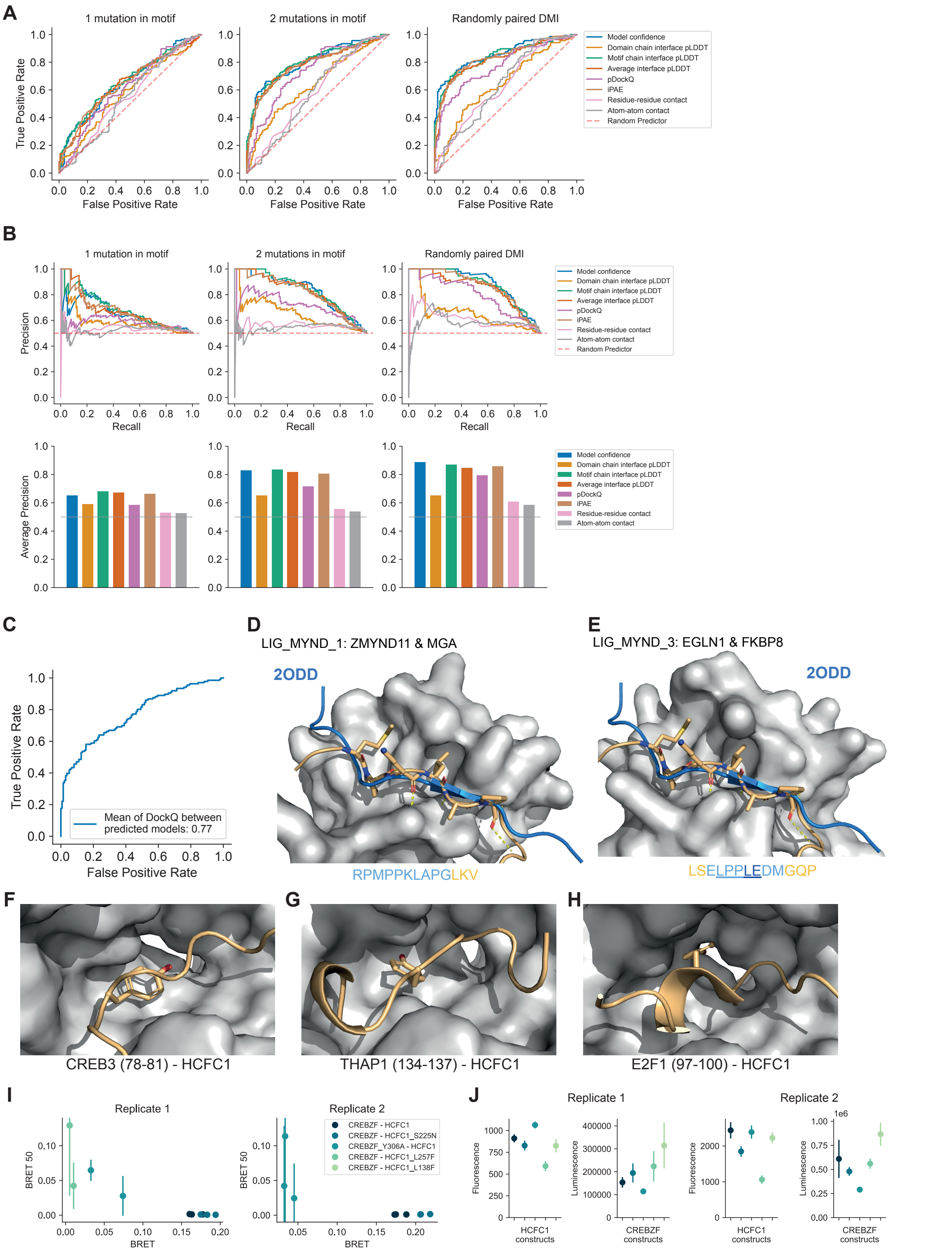

Appendix Figure S2. Benchmarking and application of AF for DMI interface prediction using minimal interacting fragments.

A Receiver operating characteristic (ROC) curve of various metrics extracted from AF models when using the DMI benchmark dataset as the positive reference and the following

sets as random reference: Left, 1 mutation introduced in conserved motif position; middle, 2 mutations introduced in conserved motif positions, right, randomly shuffled domain-motif pairs. **B** Precision recall curve of various metrics determined for benchmark datasets as in A. **C** ROC curve of mean DockQ between the top five AF structural models returned for a given input, assessed using the DMI positive reference set and random pairings of domains and motifs as in A. The AUROC of the metric is indicated in the legend of the ROC curve. **D-E** Superimposition of AF structural model for motif class LIG_MYND_1 (D) and LIG_MYND_3 (E) (orange) with homologous solved structures (PDB:2ODD) from motif class LIG_MYND_2 (blue). The motif sequence used for prediction is indicated at the bottom, colored by pLDDT (dark blue=highest pLDDT). **F-H** AF models for three motif instances (orange) of LIG_HCF-1_HBM_1 predicted to bind into a pocket on the Kelch domain of HCFC1 (gray). Motif positions are indicated below the figures. The key tyrosines of the motif sequences are drawn as sticks. **I** BRET50 estimates from fitting titration curves shown in Fig 1G are plotted vs. BRET values that were corrected for bleedthrough and measured at a 2:50 ng DNA transfection ratio for wildtype and mutant CREBZF-HCFC1 pairs. Error bars indicate the standard error. Data is shown for two technical replicates for the first biological replicate and three technical replicates for the second biological replicate. **J** Fluorescence and total luminescence are shown for wildtype and mutant CREBZF-HCFC1 pairs measured at a 2:50 ng DNA transfection ratio. Error bars indicate STD of two technical replicates for the first biological replicate and three technical replicates for the second biological replicate. Coloring as in I.

Figure S3

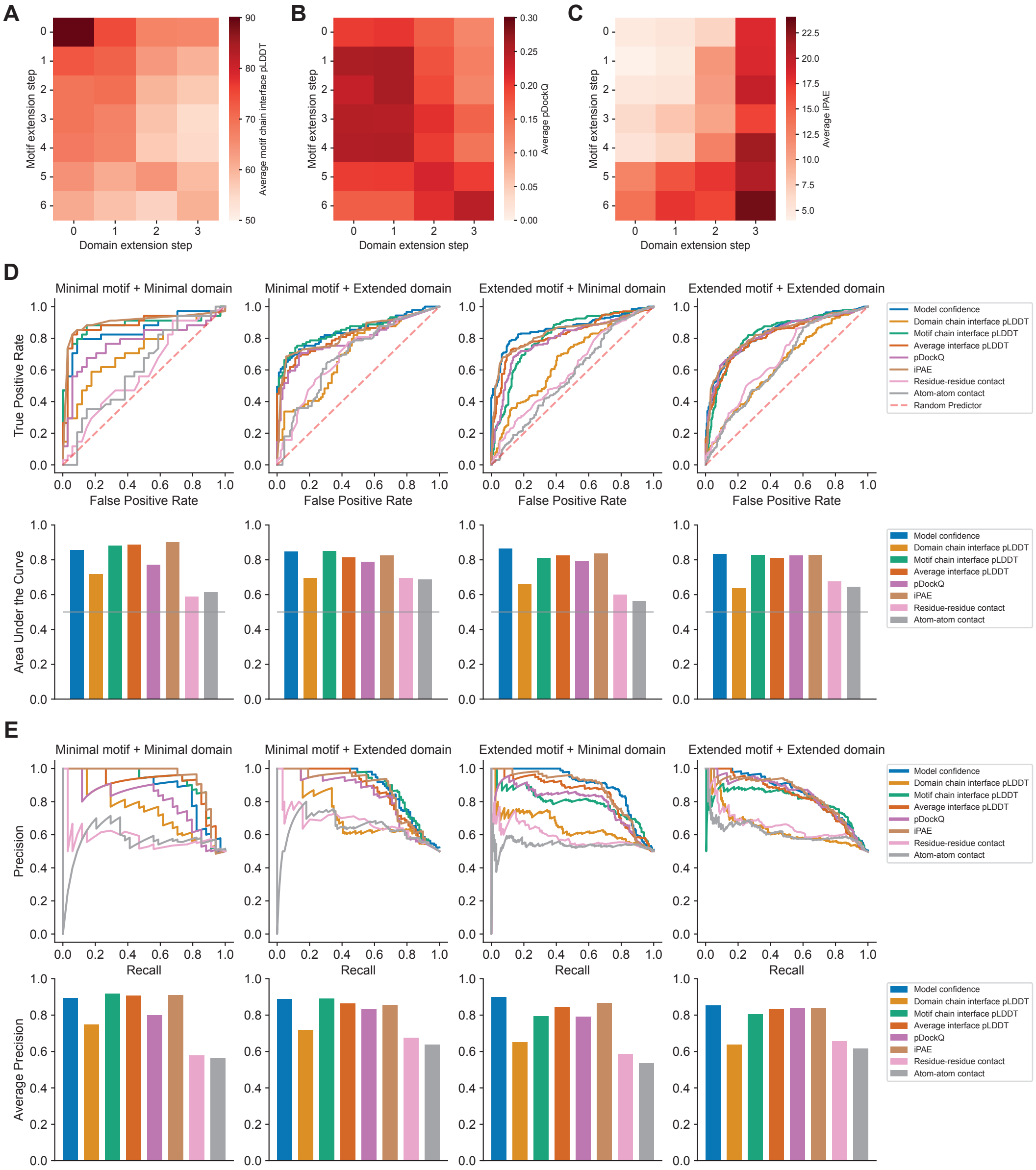

Appendix Figure S3. Effect of protein fragment extensions on the accuracy of AF predictions.

A-C Heatmap of the average motif interface pLDDT (A), pDockQ (B), and iPAE (C) for combinations of different motif and domain sequence extensions using a positive reference set consisting of 31 DMI structures. Extensions like in Fig 2A. **D** ROC curves (top) and corresponding AUROC values (bottom) of various metrics extracted from AF models when using the DMI extension dataset split by different combinations of motif and domain extensions as indicated on the top of each graph. Gray horizontal line indicates the AUROC of a random predictor. **E** Precision recall curves (top) and area under the precision recall curve as quantified by average precision (bottom) for various metrics extracted from AF models determined for benchmark datasets as in D.

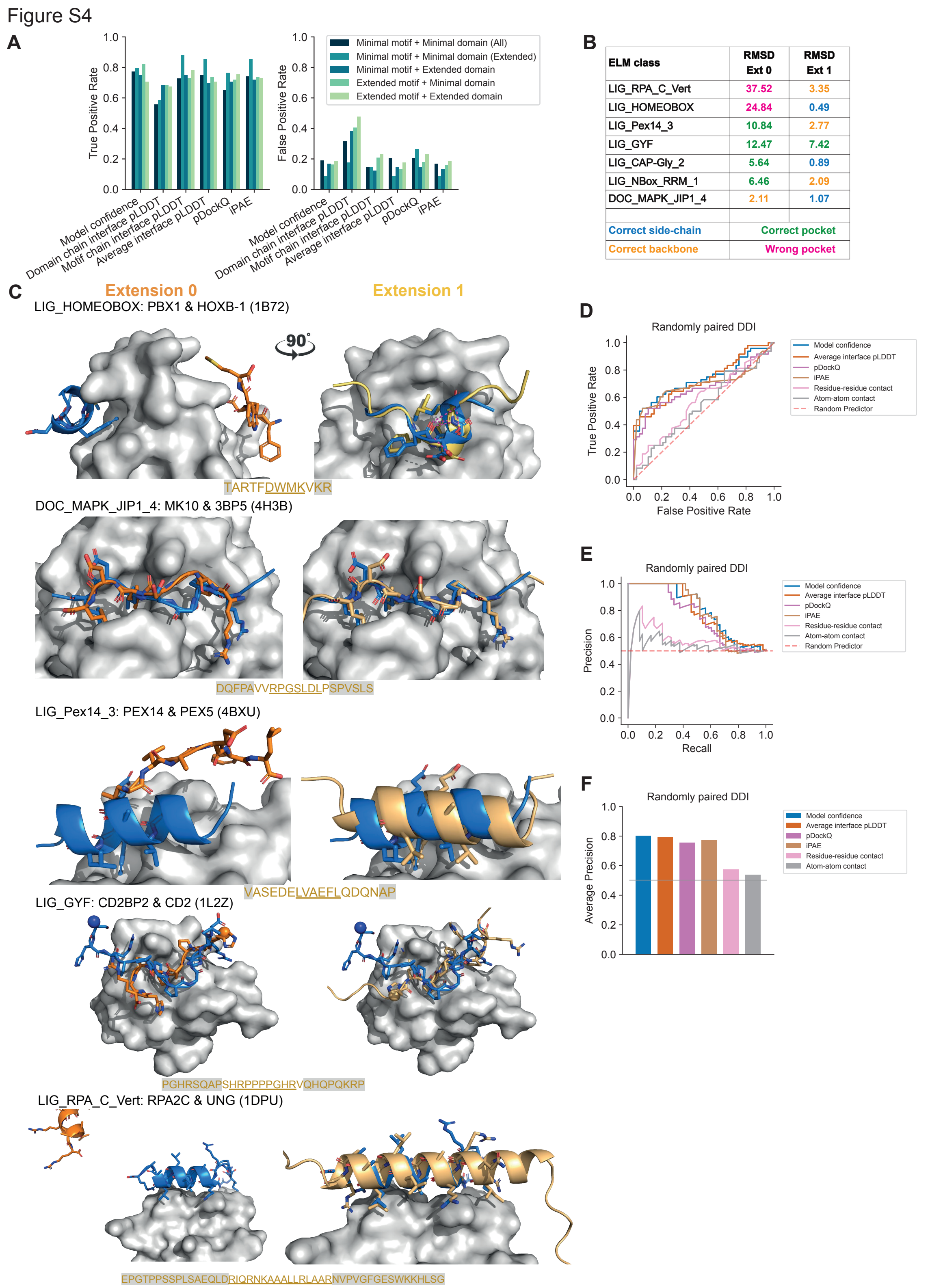

ELM class	RMSD Ext 0	RMSD Ext 1
LIG_RPA_C_Vert	37.52	3.35
LIG_HOMEOBOX	24.84	0.49
LIG_Pex14_3	10.84	2.77
LIG_GYF	12.47	7.42
LIG_CAP-Gly_2	5.64	0.89
LIG_NBox_RRM_1	6.46	2.09
DOC_MAPK_JIP1_4	2.11	1.07
Correct side-chain	Correct pocket	
Correct backbone	Wrong pocket	

Appendix Figure S4. Effect of protein fragment extensions on the accuracy of AF predictions.

A True and false positive rate (left and right, respectively) based on optimal cutoffs from Fig 2D derived for different metrics from ROC analysis for benchmarking AF with different motif

and domain extensions from the reference dataset illustrated in Fig 2A and random pairings of domain and motif sequences. **B** Table indicating the motif RMSD achieved when using minimal (extension 0) or extended motif sequences for structure prediction for all inspected motif extension cases. Extension 1 refers to extension of the minimal motif sequence by the length of the motif to the left and right. Color coding indicates the accuracy classes of the respective structural models as shown in Fig 1A. **C** Superimposition of the structural model of the minimal (left, orange) or extended (right, yellow) motif sequence with the solved structure (motif in blue) for five different motif classes as indicated on the top of each panel. The motif sequence from the solved structure is indicated at the bottom of each panel. Motif residues are underlined, motif residues not resolved in the structure have a gray background. Sticks indicate the motif residues, domain surfaces are shown in gray based on experimental structures. **D** ROC curves of different metrics using the DDI benchmark dataset as positive reference and random shuffling of domain-domain pairs as negative reference. **E** Precision recall curves of different metrics extracted from AF models determined for benchmark datasets as in D. **F** Area under the precision recall curve as quantified by average precision for metrics extracted from AF models determined for benchmark datasets as in D. Gray horizontal line indicates the average precision of a random predictor.

Figure S5

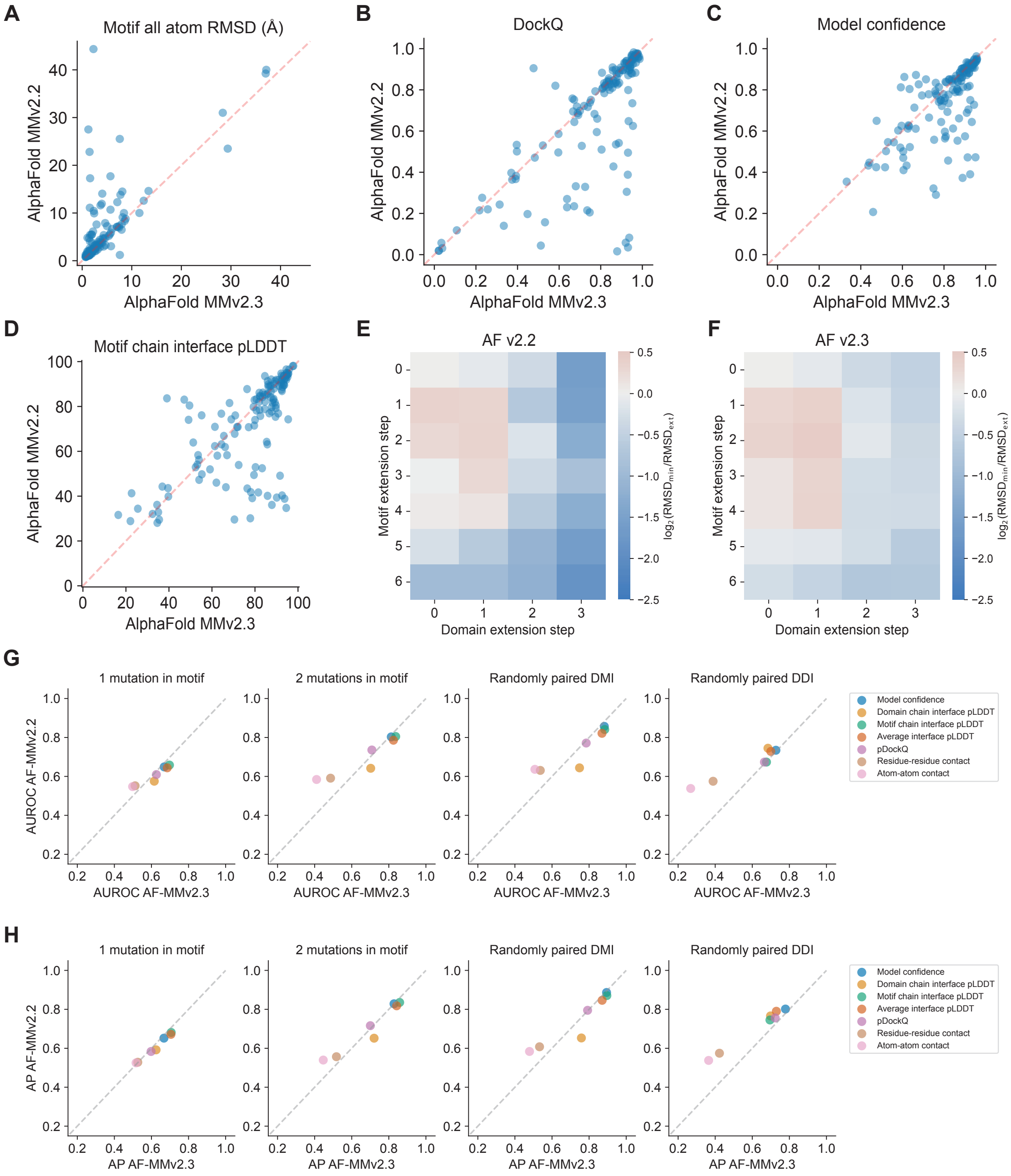

Appendix Figure S5. Comparison of AF v2.2 and v2.3 prediction performance.
A Scatterplot showing the motif RMSD obtained from structural models computed either with AF v2.2 or AF v2.3 using the minimal interacting regions of all annotated DMIs. **B-D** Scatterplots computed as in A showing the DockQ (B), model confidence (C), and motif chain interface pLDDT (D) for both AF versions. **E-F** Heatmaps showing the fold change in motif RMSD obtained for structural models from AF v2.2 (E) and AF v2.3 (F) upon domain or/and motif sequence extension compared to when using minimal interacting regions. Positive values indicate improved predictions from extension and negative values indicate worse prediction outcomes. **G** Scatterplots showing the AUROC obtained for different metrics derived from structural models from benchmarking AF v2.2 and AF v2.3 using the minimal interacting regions of all annotated DMIs or DDIs as the positive reference dataset and different random reference datasets: Left (DMI), 1 mutation introduced in conserved

motif position; middle-left (DMI), 2 mutations introduced in conserved motif positions, middle-right (DMI), randomly shuffled domain-motif pairs; right (DDI), randomly shuffled domain-domain pairs. Corresponding ROC curves for AF v2.2 and AF v2.3 are shown in Fig. S2A, S4D, and S6A. **H** Scatterplots as in G plotting the average precision (AP) obtained from PR curves from the same analysis as in G. Corresponding PR curves for AF v2.2 and AF v2.3 are shown in Fig S2B, S4E and S6B.

Figure S6

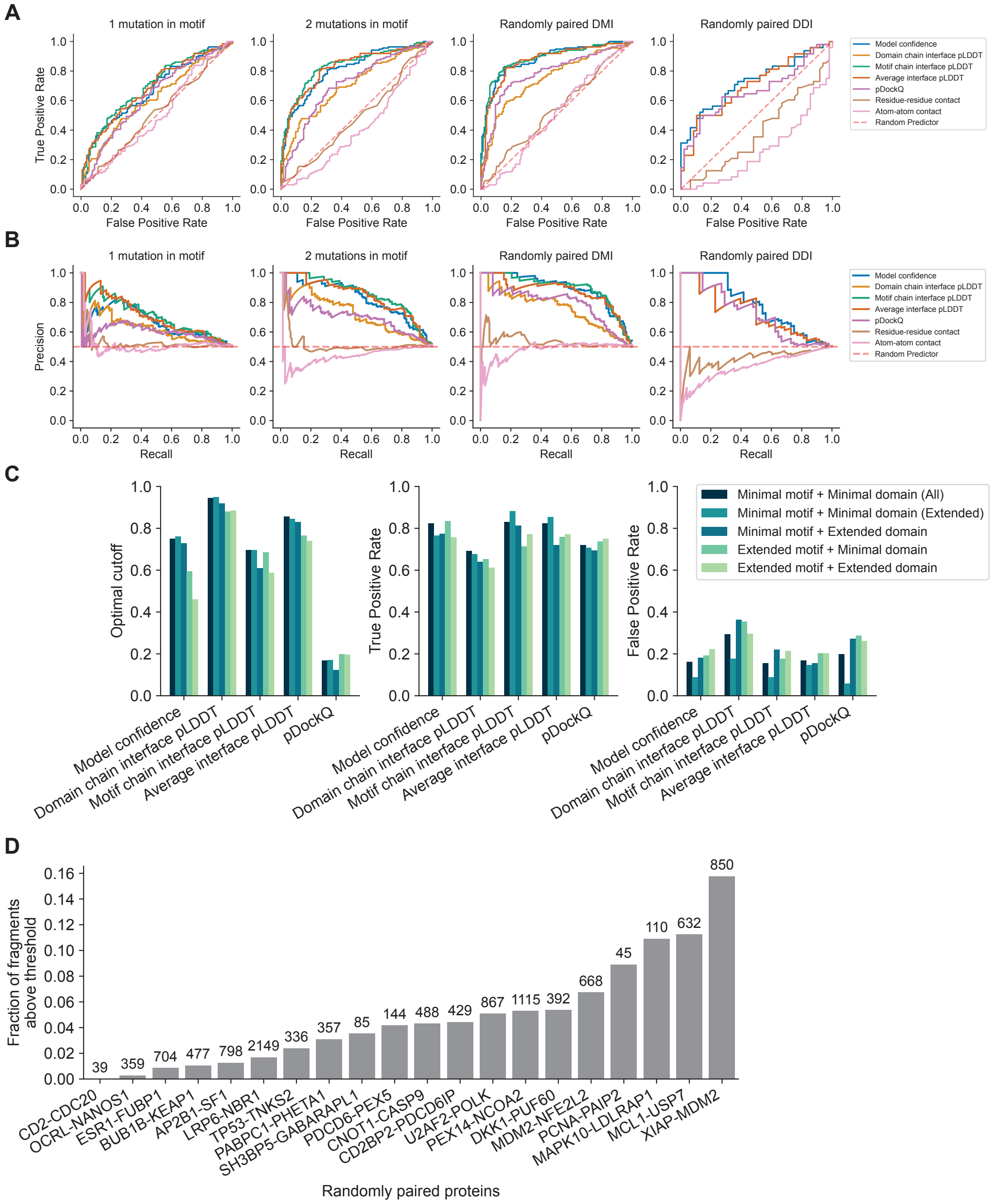

Appendix Figure S6. Performance of different metrics derived from structural models when benchmarking AF v2.3 for DMI predictions.

A ROC curves obtained for different metrics derived from structural models from benchmarking AF v2.3 using the minimal interacting regions of all annotated DMIs or DDIs as the positive reference dataset and different random reference datasets: Left (DMI), 1 mutation introduced in conserved motif position; middle-left (DMI), 2 mutations introduced in conserved motif positions, middle-right (DMI), randomly shuffled domain-motif pairs; right (DDI), randomly shuffled domain-domain pairs. **B** PR curves computed for the same datasets and AF version as in A. **C** Optimal cutoff, true, and false positive rate derived for different metrics from ROC analysis for benchmarking AF v2.3 with different motif and domain extensions from the reference dataset used in Fig 2A and randomly shuffled domain

-motif pairs. **D** Fraction of fragment pairs with structural models scoring above thresholds for 20 randomly shuffled domain-motif pairs. Numbers on top of the bars indicate the total number of fragment pairs submitted for interface prediction to AF for each random protein pair.

Figure S7

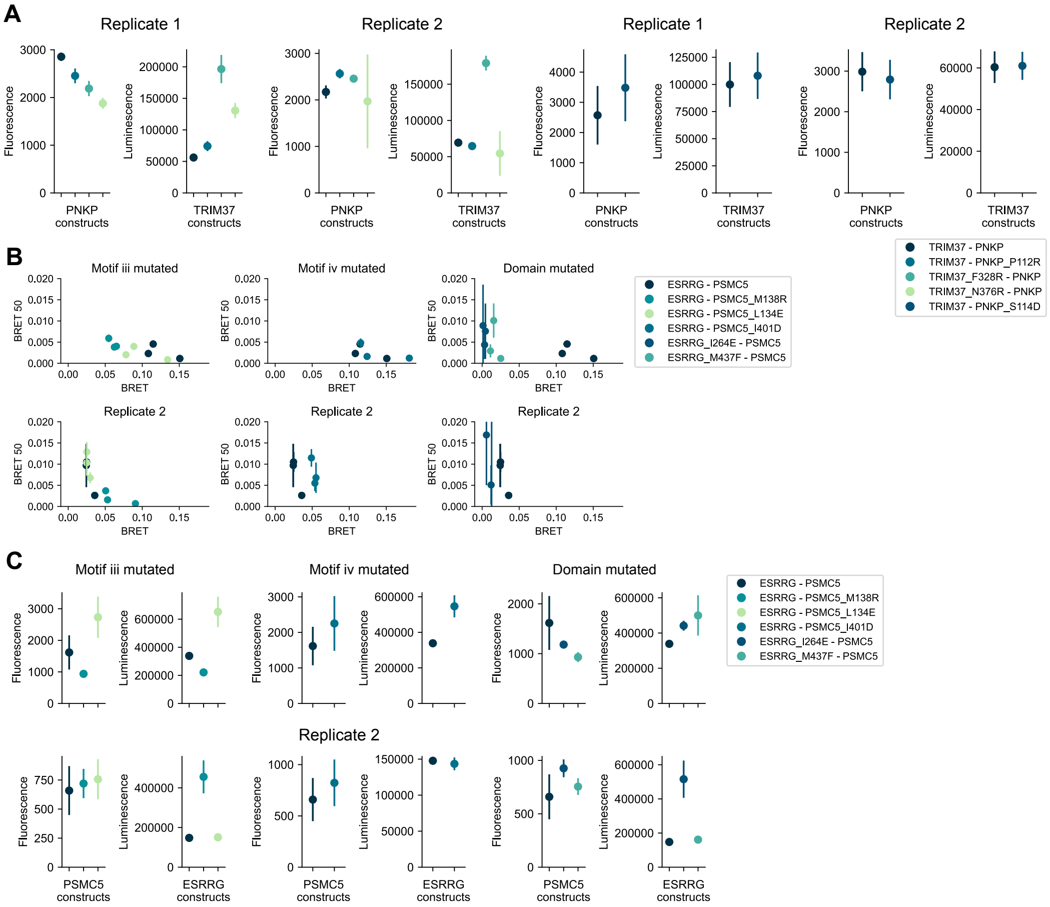

Appendix Figure S7. Expression and BRET50 plots for TRIM37-PNKP and ESRRG-PSMC5.

A Fluorescence and total luminescence are shown for wildtype and mutant TRIM37-PNKP pairs measured at a 2:50 ng DNA transfection ratio. Error bars indicate STD of three technical replicates. Data is shown for two biological replicates. **B** BRET50 estimates from fitting titration curves shown in Fig 4H are plotted vs. BRET values that were corrected for bleedthrough and measured at a 2:50 ng DNA transfection ratio for wildtype and mutant ESRRG-PSMC5 pairs. Error bars indicate the standard error. Data is shown for three technical replicates for two biological replicates each. BRET50 estimates for the second biological replicate for the ESRRG_M437F-PSMC5 pair were omitted from the graph because they exceeded the upper y-axis limit. Roman labels refer to interfaces shown in Fig 4E. **C** Fluorescence and total luminescence are shown for wildtype and mutant ESRRG-PSMC5 pairs measured at a 2:50 ng DNA transfection ratio. Error bars indicate STD of three technical replicates. Data is shown for two biological replicates.

Figure S8

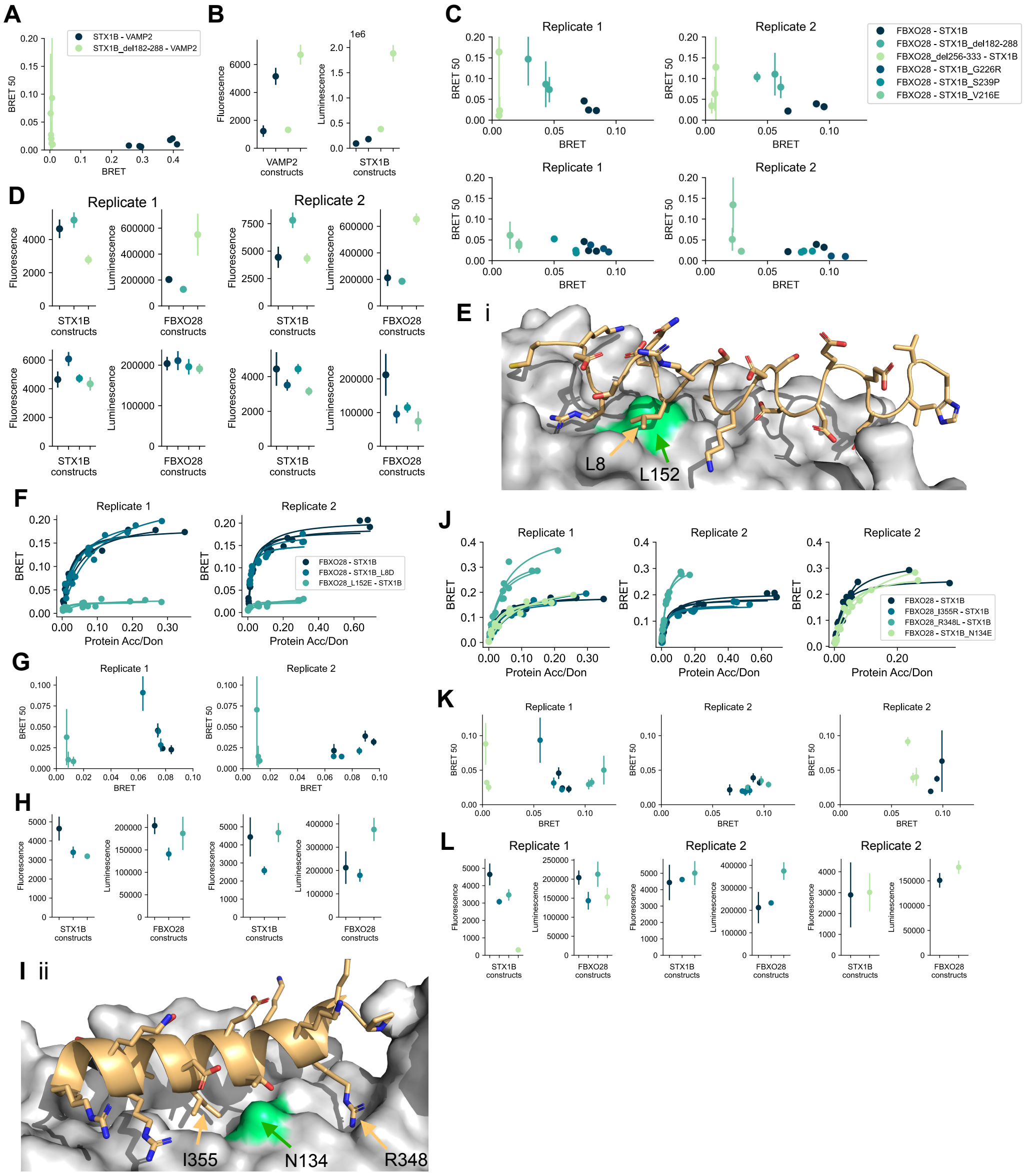

Appendix Figure S8. Structural models, expression, and BRET50 plots for STX1B-FBXO28 and STX1B-VAMP2.

A BRET50 estimates from fitting titration curves shown in Fig 5C are plotted vs. BRET values that were corrected for bleedthrough and measured at a 2:50 ng DNA transfection ratio for wildtype and mutant STX1B-VAMP2 pairs. Error bars indicate the standard error. Data is shown for three technical replicates for two biological replicates each. **B** Fluorescence and total luminescence are shown for wildtype and mutant STX1B-VAMP2 pairs measured at a 2:50 ng DNA transfection ratio. Error bars indicate STD of three technical replicates. Data is shown for two biological replicates. **C** Data shown as in A for wildtype and mutant FBXO28-STX1B pairs relating to interface iii (Fig 5A,D). **D** Data shown as in B for wildtype and mutant FBXO28-STX1B pairs shown in C. **E** Structural model corresponding to interface i shown in Fig 5A. Mutated residues on the domain (green) and motif side are labeled. **F** BRET titration curves are shown for wildtype and mutant FBXO28-STX1B pairs relating to interface i shown in E with two biological replicates, each with three

technical replicates. Protein acceptor over protein donor expression levels are plotted on the x-axis determined from fluorescence and luminescence measurements, respectively. **G** Data shown as in A for wildtype and mutant FBXO28-STX1B pairs relating to interface i. **H** Data shown as in B for wildtype and mutant FBXO28-STX1B pairs relating to interface i. **I** Structural model corresponding to interface ii shown in Fig 5A. Mutated residues on the domain (green) and motif side are labeled. **J** Data shown as in F for wildtype and mutant FBXO28-STX1B pairs relating to interface ii. **K** Data shown as in A for wildtype and mutant FBXO28-STX1B pairs relating to interface i. **L** Data shown as in B for wildtype and mutant FBXO28-STX1B pairs relating to interface i.

Figure S9

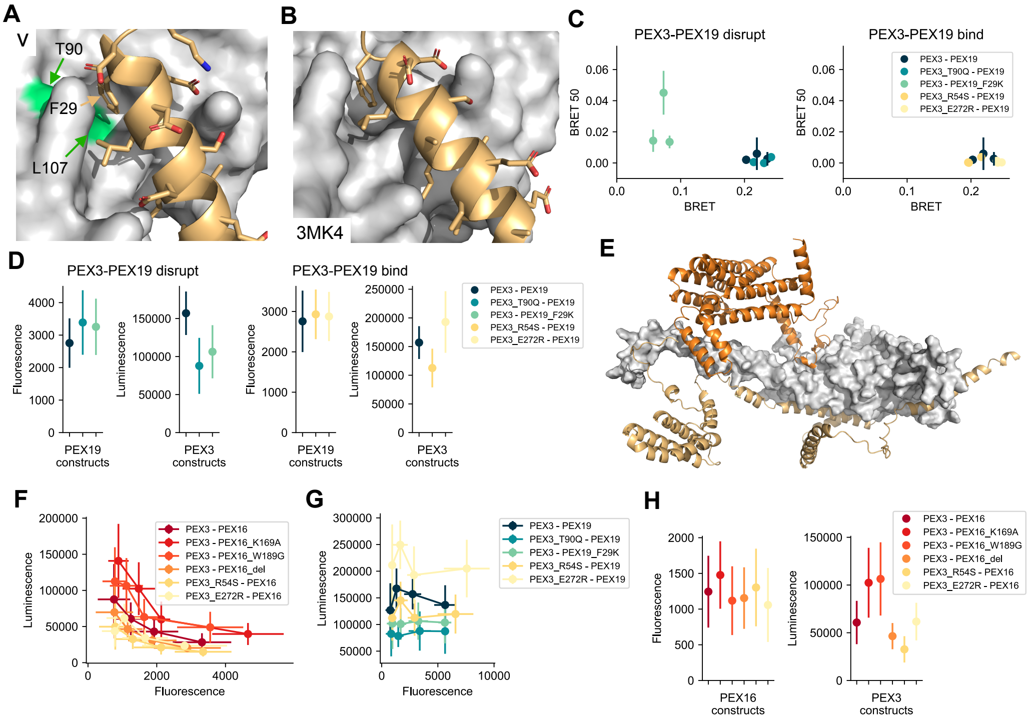

Appendix Figure S9. Structural models, expression, and BRET50 plots for PEX3-PEX19 and PEX3-PEX16.

A Structural model of PEX3-PEX19 corresponding to interface v as shown in Fig 5G. Mutated residues on the domain (green) and motif side are labeled. **B** Structure from PDB:3MK4 showing the PEX19 N-terminal motif bound to the PEX3 domain. **C** BRET50 estimates from fitting titration curves shown in Fig 5H are plotted vs. BRET values that were corrected for bleedthrough and measured at a 2:50 ng (for PEX3 and PEX3_T90Q) or 8:50 ng (for PEX3, PEX3_R54S, PEX3_E272R) DNA transfection ratio for wildtype and mutant PEX3-PEX19 pairs. Error bars indicate the standard error. Data is shown for three technical replicates. The left panel corresponds to mutant constructs that should disrupt binding while mutants shown in the right panel were aimed to disrupt binding to PEX16 and thus should not disrupt binding to PEX19. **D** Fluorescence and total luminescence are shown for wildtype and mutant PEX3-PEX19 pairs measured at a 2:50 or 8:50 ng DNA transfection ratio (see panel C). Error bars indicate STD of three technical replicates. **E** Structural model obtained with AF for the trimeric complex of PEX3 (gray), PEX19 (yellow), and PEX16 (orange) using full length sequences as input. **F** PEX3 expression levels measured in luminescence units plotted for co-transfections with increasing PEX16 protein amounts measured in fluorescence units. Error bars indicate STD of three technical replicates. **G** PEX3 expression levels measured in luminescence units plotted for co-transfections with increasing PEX19 protein amounts measured in fluorescence units. Error bars indicate STD of three technical replicates. **H** Data shown as in D for wildtype and mutant constructs of PEX3-PEX16 pairs. Measures are taken for 2:25 ng DNA transfection ratios.

Figure S10

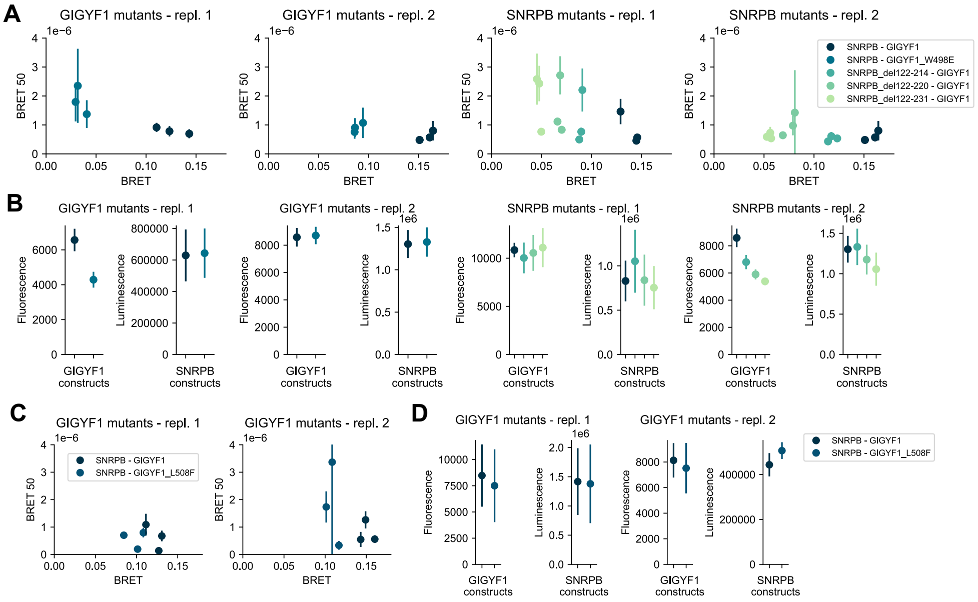

Appendix Figure S10. Expression and BRET50 plots for SNRPB-GIGYF1.

A BRET50 estimates from fitting titration curves shown in Fig 6D are plotted vs. BRET values that were corrected for bleedthrough and measured at a 2:50 ng DNA transfection ratio for wildtype and mutant SNRPB-GIGYF1 pairs. Error bars indicate the standard error. Data is shown for three technical replicates for two biological replicates each. **B** Fluorescence and total luminescence are shown for wildtype and mutant SNRPB-GIGYF1 pairs measured at a 2:50 ng DNA transfection ratio. Error bars indicate STD of three technical replicates. Data is shown for two biological replicates. Coloring as in A. **C** Data shown as in A for wildtype and mutant SNRPB-GIGYF1 pairs fitted from titration curves shown in Fig 6E. **D** Data shown as in B for wildtype and mutant SNRPB-GIGYF1 pairs shown in C.
