# Supplementary figures and images for: Systematic discovery of protein interaction interfaces using AlphaFold and experimental validation
Source: Mol Syst Biol. 2024 Jan 15;20(2):75–97. doi: 10.1038/s44320-023-00005-6 (PMC10883280; doi:10.1038/s44320-023-00005-6)

Raw data

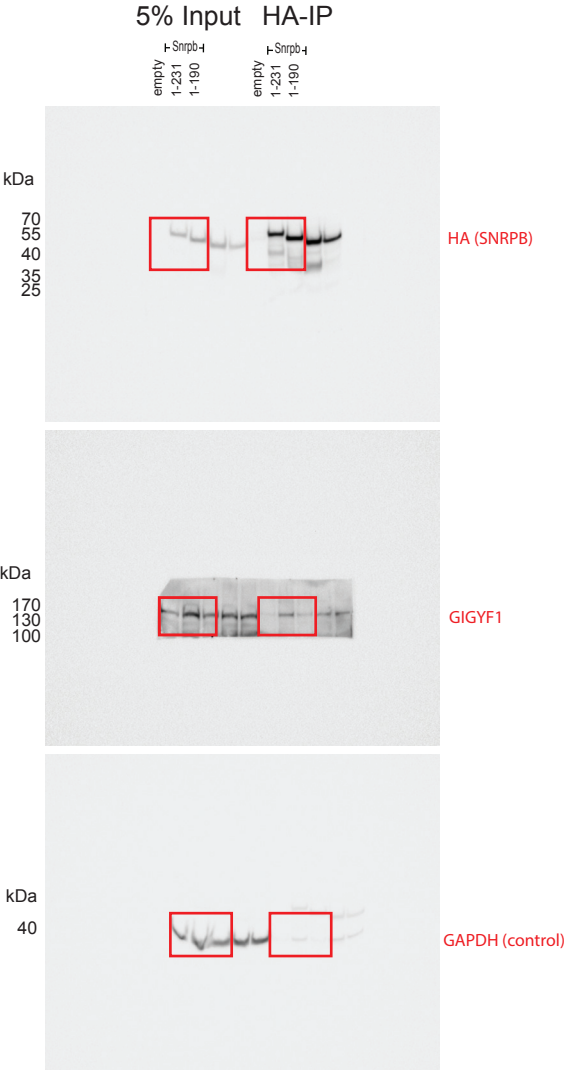

Figure 6G

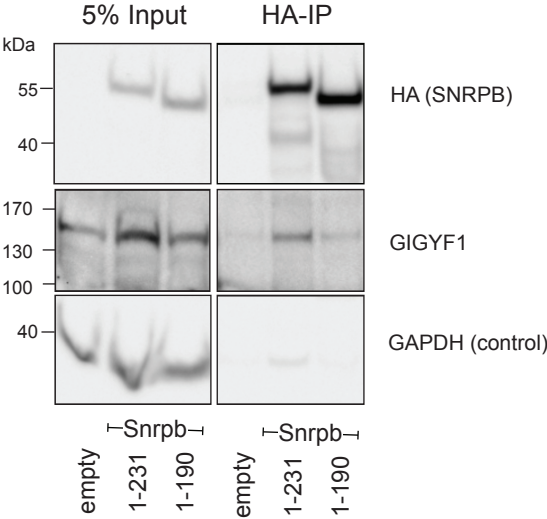

Supplement: Supplementary file 18 — Source Data Fig. 5 [file 44320_2023_5_MOESM18_ESM.zip › Figure 6/6F/western_blot_cropping_label.pdf]

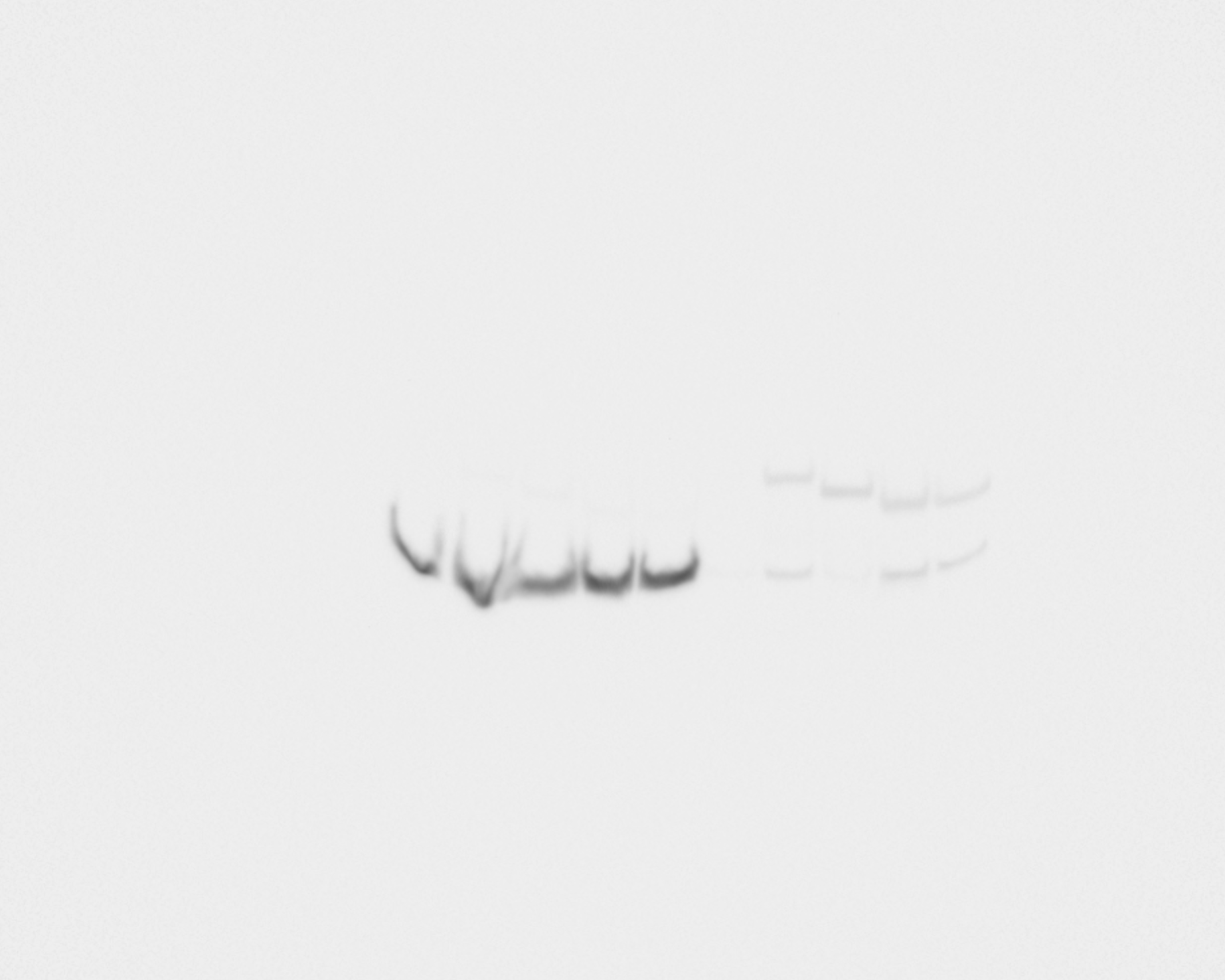

Supplement: Supplementary file 18 — Source Data Fig. 5 [file 44320_2023_5_MOESM18_ESM.zip › Figure 6/6F/GAPDH/G20 Lab 2023-07-28 12h01m23s.tif]

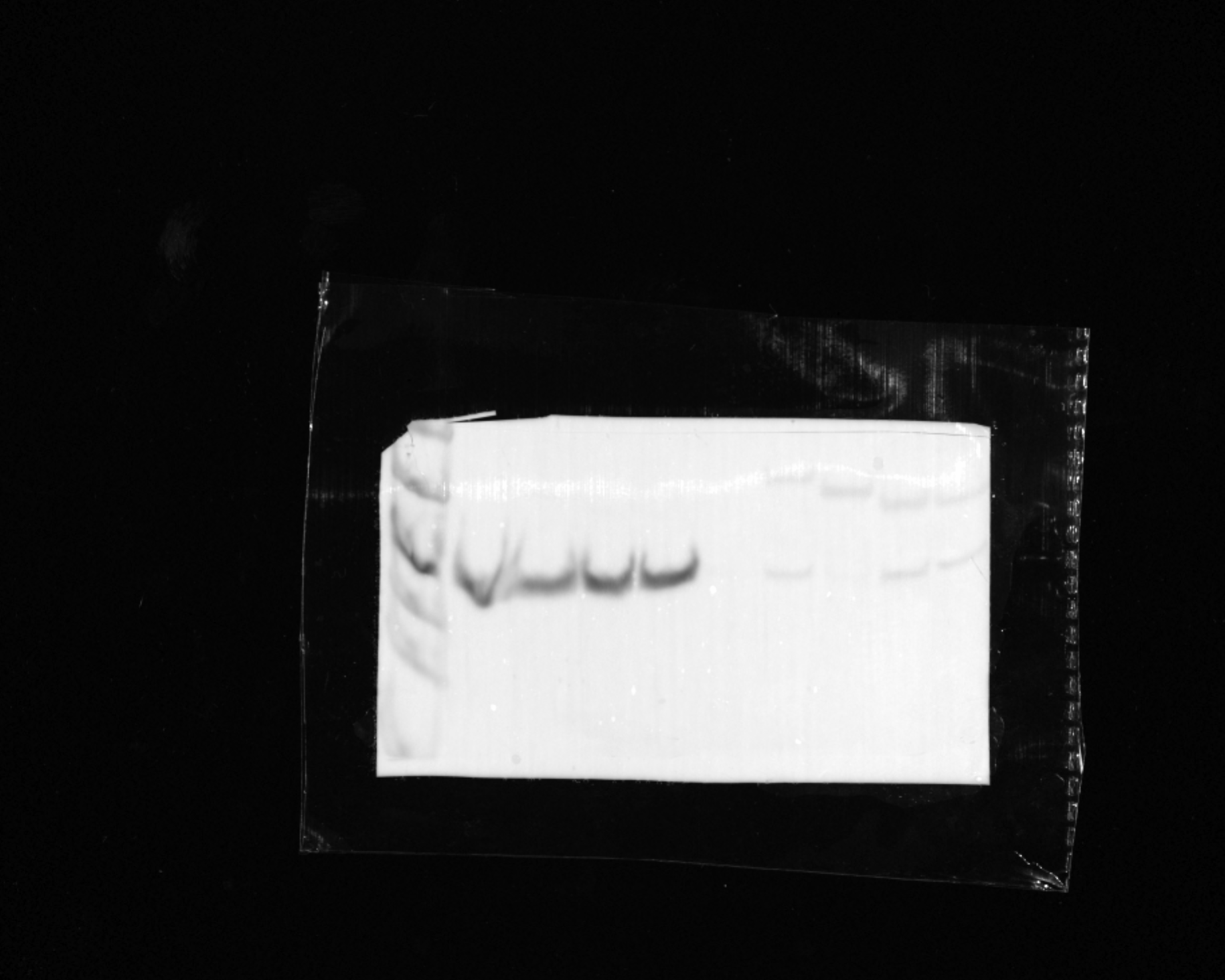

Supplement: Supplementary file 18 — Source Data Fig. 5 [file 44320_2023_5_MOESM18_ESM.zip › Figure 6/6F/GAPDH/G20 Lab 2023-07-28 12h01m23s+G20 Lab 2023-07-28 12h00m30s.tif]

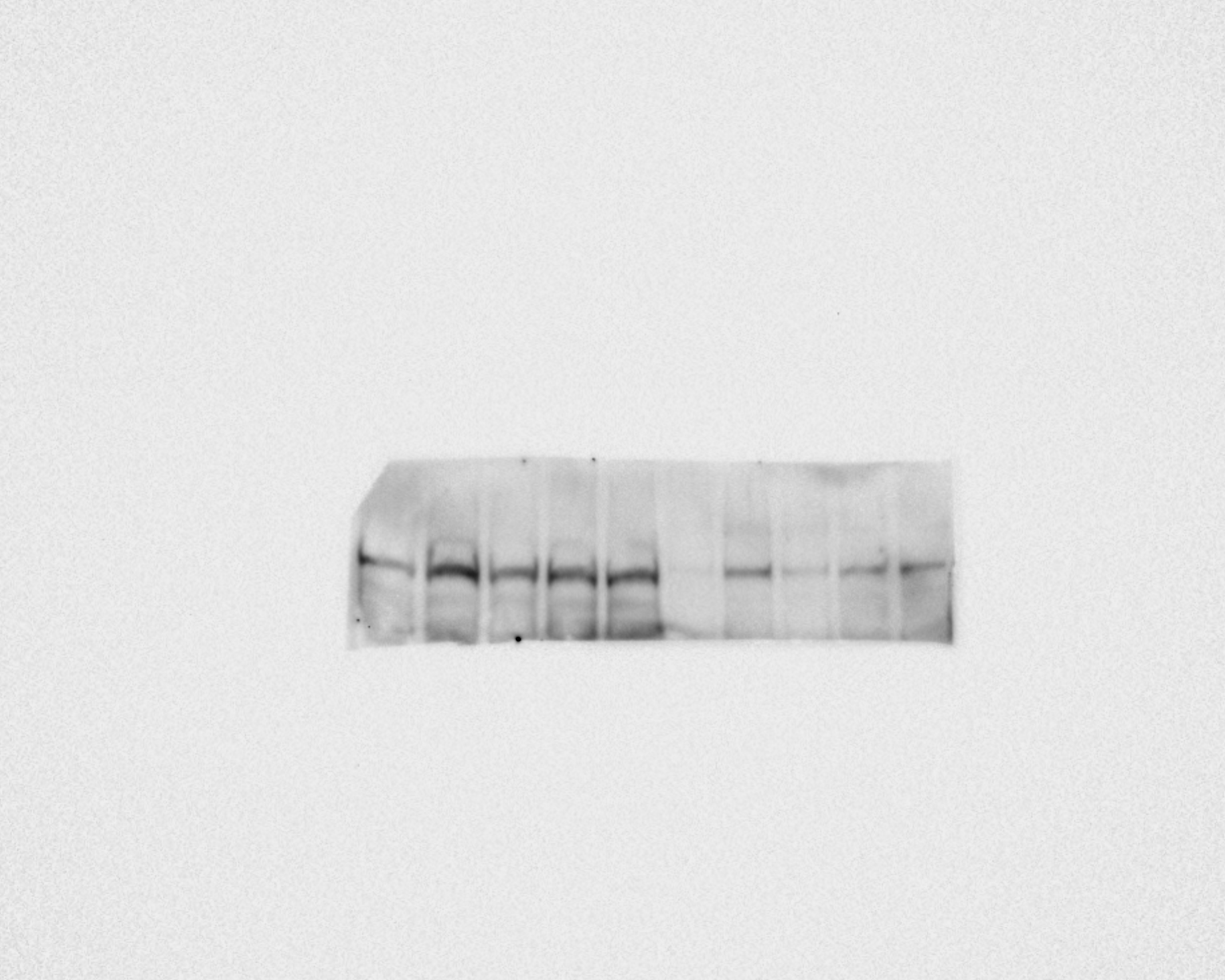

Supplement: Supplementary file 18 — Source Data Fig. 5 [file 44320_2023_5_MOESM18_ESM.zip › Figure 6/6F/GIGYF1/G20 Lab 2023-07-28 09h34m42s.tif]

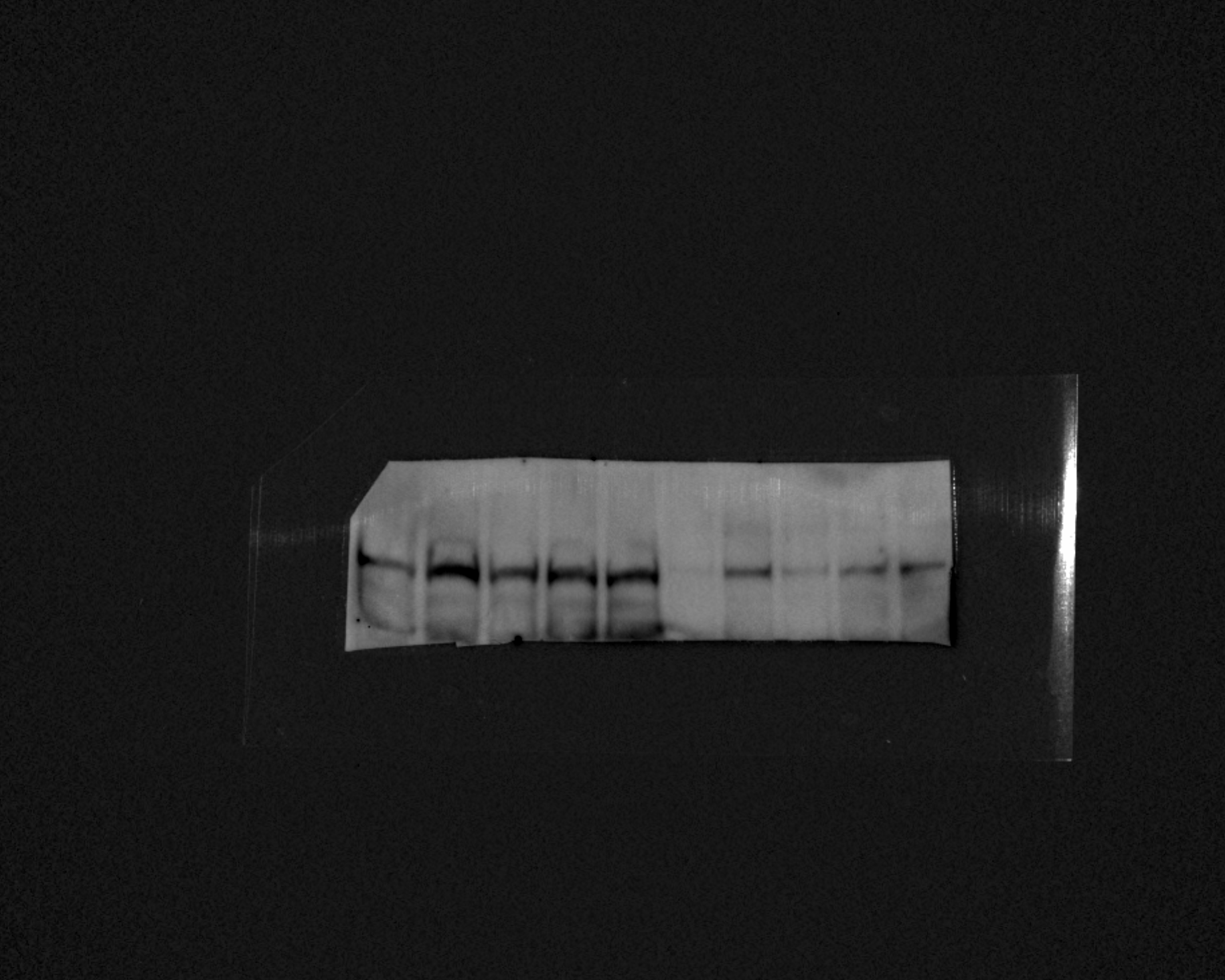

Supplement: Supplementary file 18 — Source Data Fig. 5 [file 44320_2023_5_MOESM18_ESM.zip › Figure 6/6F/GIGYF1/G20 Lab 2023-07-28 09h34m42s+G20 Lab 2023-07-28 09h36m17s.tif]

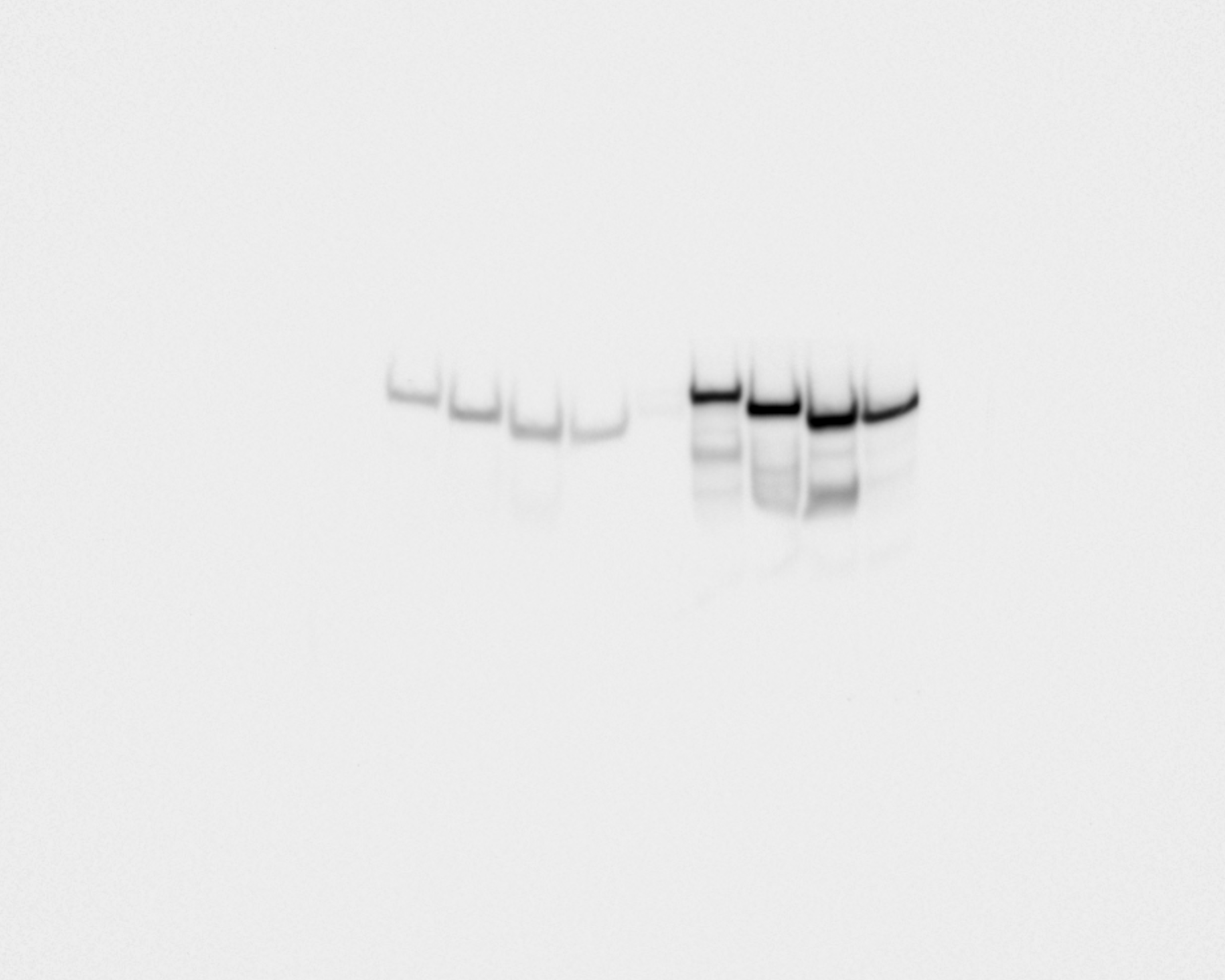

Supplement: Supplementary file 18 — Source Data Fig. 5 [file 44320_2023_5_MOESM18_ESM.zip › Figure 6/6F/HA/G20 Lab 2023-07-28 09h17m47s.tif]

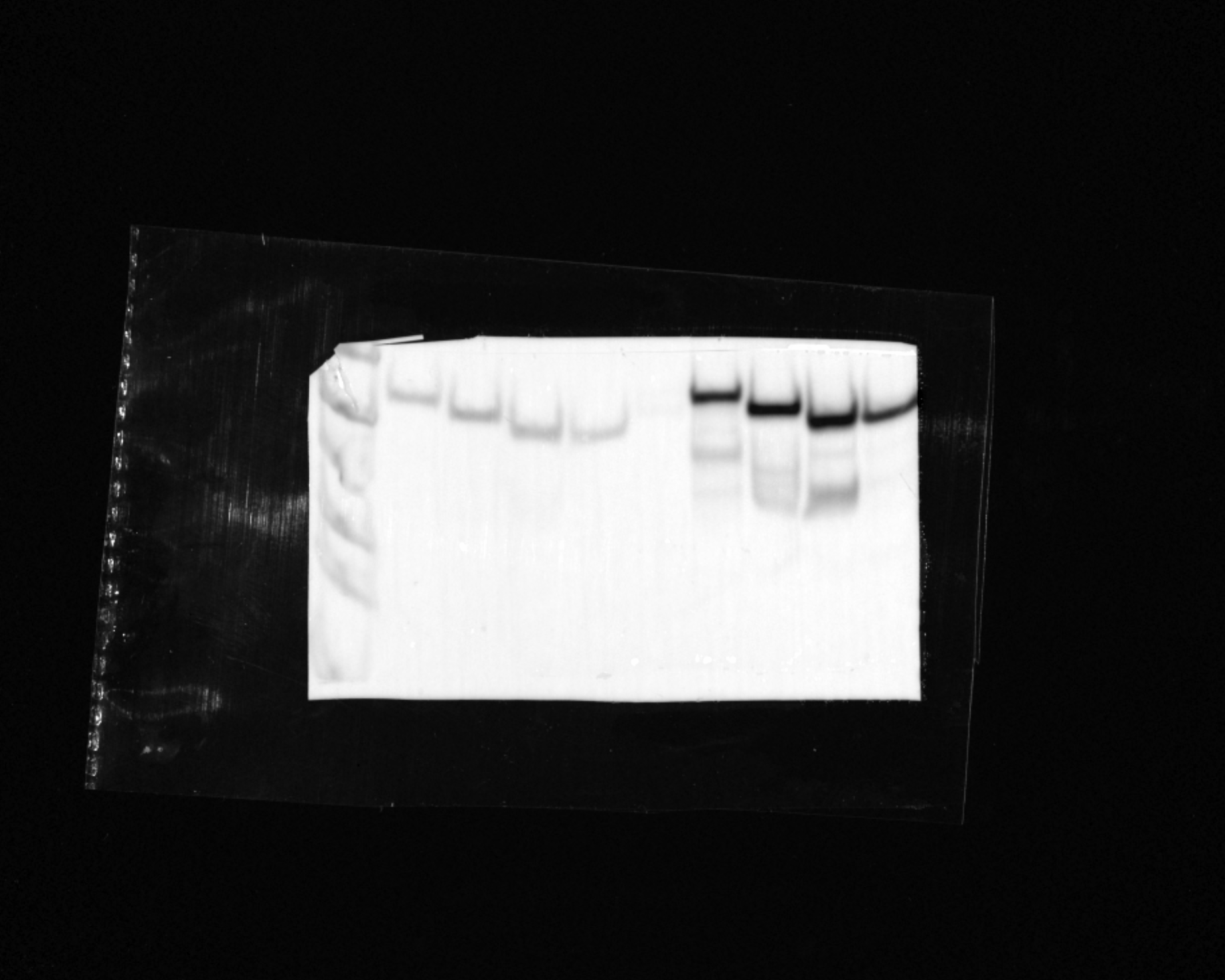

Supplement: Supplementary file 18 — Source Data Fig. 5 [file 44320_2023_5_MOESM18_ESM.zip › Figure 6/6F/HA/G20 Lab 2023-07-28 09h17m47s+G20 Lab 2023-07-28 09h16m30s.tif]
